# Estimation of treatment effects in short‐term depression studies. An evaluation based on the ICH E9(R1) estimands framework

**DOI:** 10.1002/pst.2214

**Published:** 2022-06-09

**Authors:** Marian Mitroiu, Steven Teerenstra, Katrien Oude Rengerink, Frank Pétavy, Kit C. B. Roes

**Affiliations:** ^1^ Methodology Working Group College ter Beoordeling van Geneesmiddelen ‐ Medicines Evaluation Board Utrecht The Netherlands; ^2^ Clinical Trial Methodology Department, Biostatistics and Research Support, Julius Center for Health Sciences and Primary Care, Biostatistics and Research Support University Medical Center Utrecht, Utrecht University Utrecht The Netherlands; ^3^ Department for Health Evidence, Section Biostatistics Radboud University Medical Center Nijmegen The Netherlands; ^4^ Data Analytics and Methods Taskforce European Medicines Agency Amsterdam The Netherlands

**Keywords:** clinical trial, estimand, intercurrent event, missing data, sensitivity analysis

## Abstract

Estimands aim to incorporate intercurrent events in design, data collection and estimation of treatment effects in clinical trials. Our aim was to understand what estimands may correspond to efficacy analyses commonly employed in clinical trials conducted before publication of ICH E9(R1). We re‐analysed six clinical trials evaluating a new anti‐depression treatment. We selected the following analysis methods—ANCOVA on complete cases, following last observation carried forward (LOCF) imputation and following multiple imputation; mixed‐models for repeated measurements without imputation (MMRM), MMRM following LOCF imputation and following jump‐to‐reference imputation; and pattern‐mixture mixed models. We included a principal stratum analysis based on the predicted subset of the study population who would not discontinue due to adverse events or lack of efficacy. We translated each analysis into the implicitly targeted estimand, and formulated corresponding clinical questions. We could map six estimands to analysis methods. The same analysis method could be mapped to more than one estimand. The major difference between estimands was the strategy for intercurrent events, with other attributes mostly the same across mapped estimands. The quantitative differences in MADRS10 population‐level summaries between the estimands were 4–8 points. Not all six estimands had a clinically meaningful interpretation. Only a few analyses would target the same estimand, hence only few could be used as sensitivity analyses. The fact that an analysis could estimate different estimands emphasises the importance of prospectively defining the estimands targeting the primary objective of a trial. The fact that an estimand can be targeted by different analyses emphasises the importance of prespecifying precisely the estimator for the targeted estimand.

## 1 INTRODUCTION

The National Research Council (US) Panel on Handling Missing Data in Clinical Trials report on missing data in clinical trials^1^ triggered new developments on missing data in academic, regulatory and industry sectors.^2^, ^3^, ^4^, ^5^, ^6^, ^7^, ^8^, ^9^, ^10^, ^11^, ^12^, ^13^, ^14^, ^15^ The report and scientific discussions informed the development of the ICH E9(R1) Addendum on estimands and sensitivity analysis in clinical trials, that was recently published.^16^ This Addendum aims to enhance the transparency and understanding of treatment effects and how they are estimated by precisely describing a priori the estimands and the associated data collection. It considers that the missing data problem can be better addressed by integrating intercurrent events in the estimation of treatment effects. Missing data are defined in the Addendum as ‘Data that would be meaningful for the analysis of a given estimand but were not collected. They should be distinguished from data that do not exist or data that are not considered meaningful because of an intercurrent event’. Intercurrent events are defined as ‘Events occurring after treatment initiation that affect either the interpretation or the existence of the measurements associated with the clinical question of interest’. The estimand is defined using five attributes: treatment, population, variable, population‐level summary and any other strategies for intercurrent events. It is important to understand the potential impact of estimands framework compared to current practice in establishing treatment effects in randomised controlled trials, both scientifically as well as from a regulatory perspective. In this paper we re‐analyse six short‐term depression trials that supported the initial marketing authorisation of mirtazapine to evaluate common analysis strategies against the new concept of estimands. The main focus is on the impact of dealing with intercurrent events. The treatment attribute defines the regimen involving a precise sequence of interventions. For the depression trials, the investigated treatment is orally administered mirtazapine (or comparator) in addition to standard of care, with dose titrated upward and a pre‐defined selection of prohibited concomitant medication. The population attribute describes the target population, in our example ‘adults suffering from depression (as defined by DSM IV diagnosis and severity cut‐offs at baseline), and not suffering from defined co‐morbidities’ (we use ‘adults suffering from depression’ thereafter). The variable attribute describes the clinical outcome to be obtained for each patient at scheduled visits. For most of the six trials, the primary outcome variable was MADRS10 total score to be obtained at baseline, at weeks 1–6 (end of trial). In this paper, the focus is on continuous outcomes. The population‐level summary for the variable provides the basis for the comparison between treatment conditions. For example, the difference in mean MADRS10 between the experimental and control arm at a pre‐planned timepoint, for example at 6 weeks after initiating treatment.

The Addendum suggests five strategies for addressing intercurrent events: treatment policy, hypothetical, composite variable, while‐on‐treatment and principal stratum. Treatment policy strategy (actively) ignores the intercurrent event, and uses the outcome irrespective of occurrence of the intercurrent event (enabling the ITT principle as defined in ICH E9^17^), provided that outcomes can exist after the intercurrent event. If the original measurement of the outcome might not exist after the intercurrent event or might not be meaningful, some of the other suggested strategies (e.g. composite variable) can enable estimation of treatment effects while preserving randomisation, as a way to implement the ITT principle. Hypothetical strategies emulate a scenario where the intercurrent event would not occur, that is for the defined clinical question, the value of the variable that would have been observed without the intercurrent event is of interest. Composite variable strategy incorporates the intercurrent event into the variable definition if the variable is composite (e.g. non‐responder imputation), or by assigning a value guided by the reason for missingness and/or its timing in the continuous outcome to reflect the intercurrent event (e.g. assign a worst outcome value from that scale or from values recorded in the control arm). While‐on‐treatment strategy uses the available outcome up to the last treatment administration, or up to the occurrence of the intercurrent event (e.g. up to rescue medication intake). Principal stratum strategy identifies the population that would have or would have not experienced a certain intercurrent event. There is an interplay between the treatment, population and variable attributes and the strategies for addressing intercurrent events. If a different outcome is chosen, the impact of intercurrent events may not stay the same (or the same event may not be an intercurrent event if it does not affect the outcome anymore). Some of these strategies can be defined at the level of a single attribute (treatment, population or variable), others at the level of a strategy for the remaining intercurrent events.^16^, ^18^

The objective of the present research was to understand what estimands correspond to common efficacy analyses as they were usually applied and are still applied at large, without making distinction between the different intercurrent events. Second, the aim was to assess empirically the impact of choosing between the various analysis methods, by comparing the estimated differences in treatment effect using these methods in short‐term depression trials with varying frequencies of the number and type of intercurrent events. In addition, we aimed to explore which methods could be useful as a sensitivity analysis.

## 2 METHODS

### 2.1 Trial data

**FIGURE 1:**
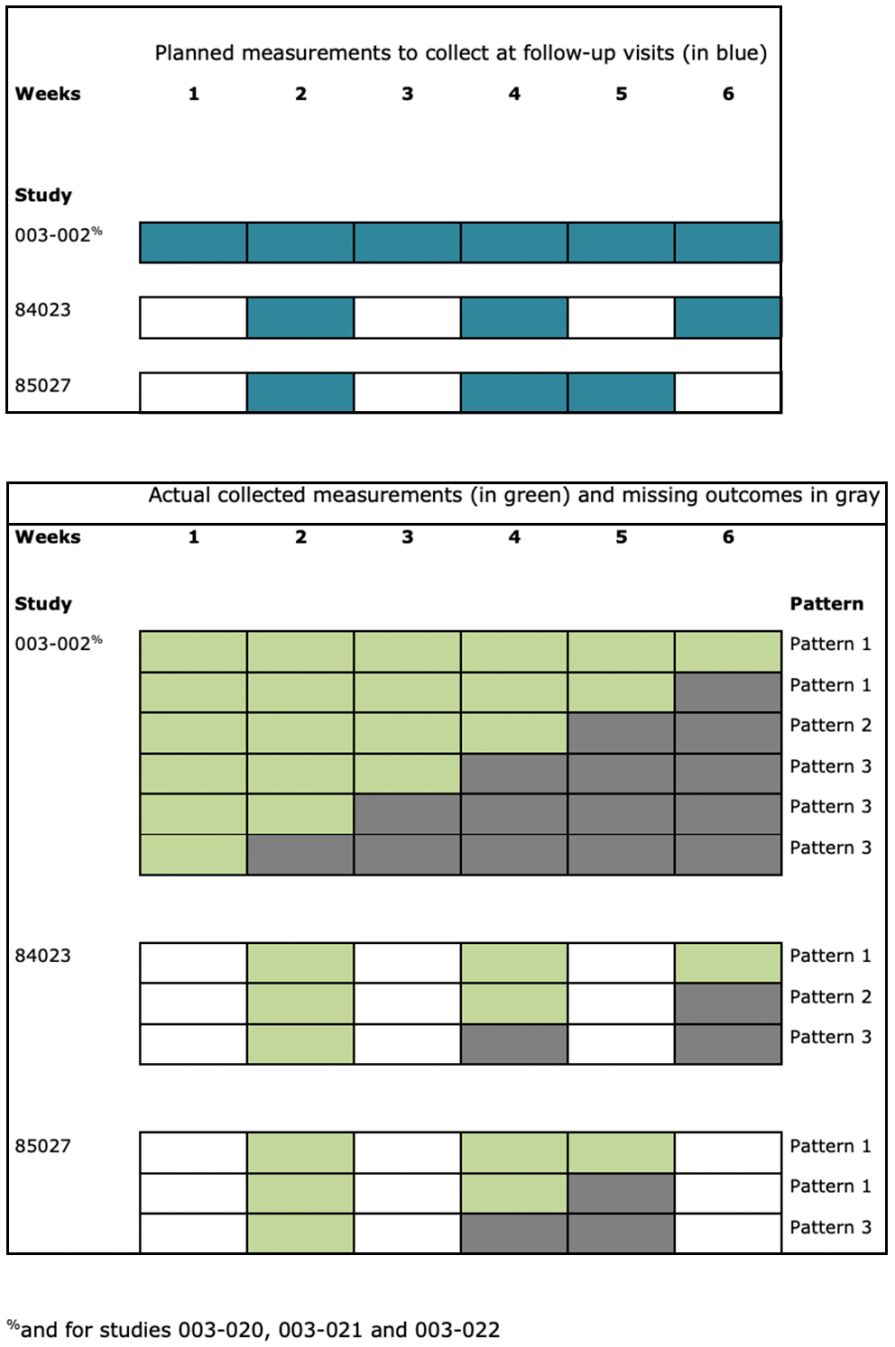
Planned follow‐up visits and patterns description. For the top and bottom panel, on the *x*‐axis are displayed the visits number at which measurements were planned to be collected. On the *y*‐axis are three studies with their corresponding design, number of visits and spacing in time. For the bottom panel, the *x*‐axis and *y*‐axis coincide with the description provided above. Additionally, on the right *y*‐axis we displayed possible patterns of observed/missing outcome data for each distinct trial design.

**FIGURE 2:**
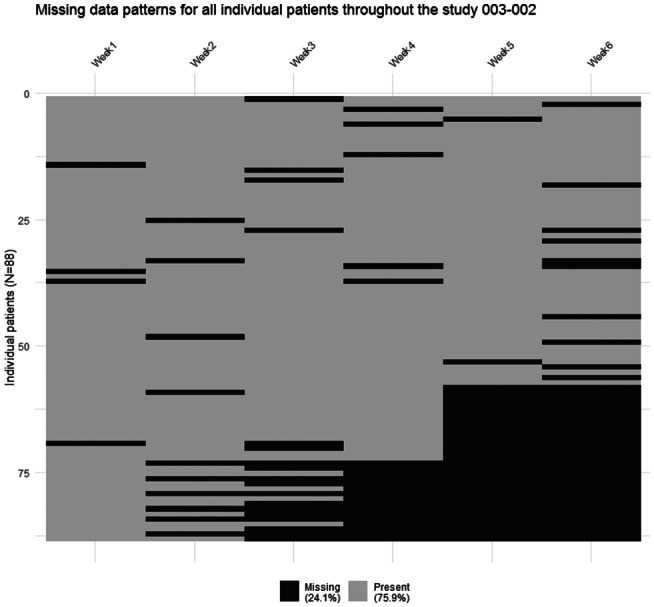
Heatmap missing data patterns for individual patients in study 003‐002. This figure displays per visit if data were present or missing for each patient randomised in that particular trial, at trial level.

**FIGURE 3:**
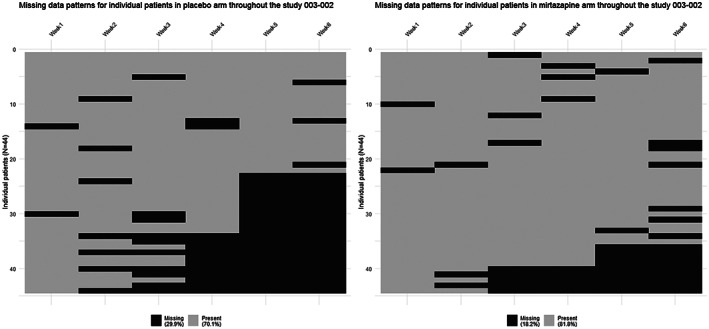
Heatmap missing data at arm level for study 003‐002. This figure displays per visit if data were present or missing for each patient randomised in that particular trial, at trial arm level.

**FIGURE 4:**
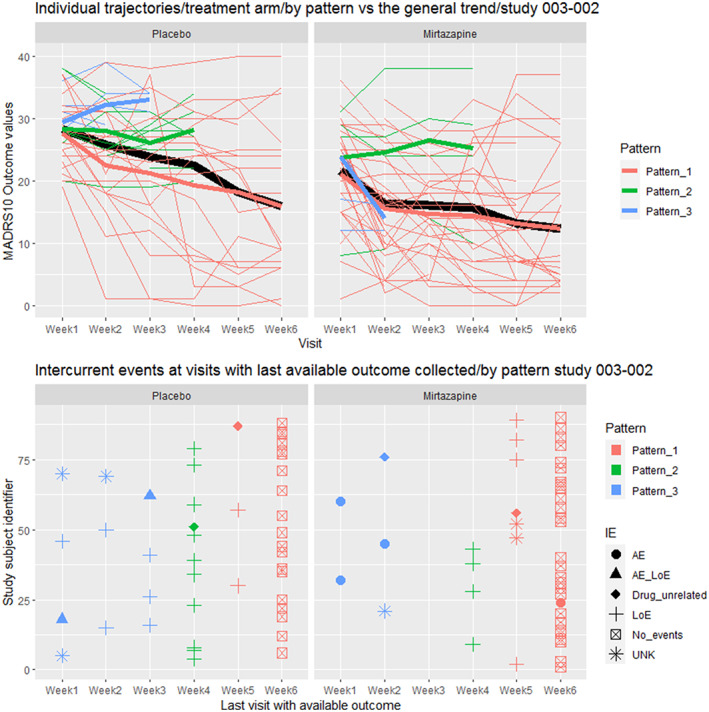
Individual trajectories of patients (Study subject identifier) by treatment arm and pattern of discontinuation with the corresponding intercurrent events. In the top panel each thin line corresponds to a patient and the observed MADRS10 throughout the trial. Each colour corresponds to a different pattern. The thick coloured lines represent the longitudinal group means for each pattern. Each thick line is the average of the thin lines. In the bottom panel the symbols correspond directly and mirror the patients' outcomes from the spaghetti plot (thin lines) from the top panel, and they match 1:1 in colour and timing with the observed outcomes and the pattern they belong to. For instance, we see in the bottom panel in the mirtazapine arm, four green ‘+’. This symbol (‘+’) stands for ‘lack of efficacy’, corresponding to the four thin green lines from the top panel. The colour green corresponds to the late dropouts pattern. The thick green line in the top panel represents the longitudinal group means and trajectory in the pattern of late dropouts. All here dropping out fortuitously due to lack of efficacy. It could be as in the placebo arm, where in the same pattern (thick green line), the patients (thin green lines) dropped out due to ‘lack of efficacy’ and ‘drug‐unrelated reasons’. The thick black line corresponds to the group means and longitudinal trajectory of all patients as observed, not differentiated by pattern or intercurrent event.

We used data from six randomised controlled trials supporting regulatory approval of a new anti‐depressive treatment (mirtazapine), designed long before ICH E9(R1).^19^ All trials were double‐blind, parallel group; three were placebo‐controlled, three were placebo‐ and active‐ controlled. Trial treatment duration and follow‐up was 5 or 6 weeks, clinical outcome was collected at baseline and at 3 or 6 timepoints post‐baseline (Figure 1). The clinical outcome of interest is MADRS10, a widely used score in depression trials, with smaller scores indicating less severe depression.^20^ Throughout the trials, patients experienced ‘intercurrent events’, causing some of the missing outcome values (Supporting Information S1) or impacting values that were observed. However, as these trials were conducted before the ICH E9(R1) era, intercurrent events of potential interest were not registered with enough level of detail. For illustrative purposes, we treated ‘study withdrawal’ as approximation of the intercurrent event of interest. Reasons for withdrawal were registered, study withdrawal coincided with stopping treatment and follow‐up, and the vast majority of reasons for withdrawal were either occurrence of an adverse event (AE) or experience of lack of efficacy (LoE). We visualised the intercurrent event patterns and outcomes of trials as follows (using study 003‐002 as example): the observed and missing data patterns with the heatmaps at trial level (Figure 2) and at arm level (Figure 3), and the longitudinal clinical outcomes, observed and missing, in conjunction with the intercurrent events corresponding to observed patterns (Figure 4). Visualisations for the other five studies are provided in Supporting Information S2. The investigator could record one or more reasons for treatment discontinuation out of the following: adverse events, lack of efficacy, insufficient compliance, efficacy, drug unrelated reasons, and unknown. The reason for discontinuation of study treatment should play a role in determining how the subsequent missing data is handled. We used one intercurrent event ‘study withdrawal’ without making explicit differentiation between the reasons that led to ‘study withdrawal’, as data collection in older trials did not allow precise enough distinction, nor did the statistical analysis in older trials distinguish between reasons for missing data. Therefore, this choice would not seriously impact the illustration of key differences between analysis models.

### 2.2 Derived estimands and comparison of analysis methods

To understand the treatment effect the methods are estimating, we derived an estimand corresponding to the analysis described, following the estimand structure and definition of the ICH E9(R1) Addendum.^16^ The depression trials were used as example for trials with longitudinally observed continuous outcomes. Relative clinical relevance of the derived estimands for depression trials is not specifically evaluated. To quantify and compare the treatment effects estimated using the selected methods, we visualised the population‐level summary of treatment effects estimated by each method with 95% CI in Forest plots. We considered a sensitivity analysis any analysis that challenges the assumptions made while targeting the same estimand. All analyses and plots were performed in RStudio software (version 1.2.5042) and relevant packages.^21^, ^22^, ^23^, ^24^, ^25^, ^26^, ^27^

### 2.3 Selection of methods

We selected common analysis methods for clinical trials (not necessarily targeted to depression trials), based on a survey,^28^ a review^29^ and on the Addendum,^16^ and included: Analysis of covariance (ANCOVA)^30^, ^31^ on complete cases (no missing data in baseline and end of trial outcomes), following last observation carried forward (LOCF) imputation,^32^ and following multiple imputation^33^; mixed‐models for repeated measurements without imputation (MMRM thereafter),^34^ MMRM following LOCF imputation and following jump‐to‐reference (J2R) imputation^35^; pattern‐mixture mixed models (PMMM),^36^, ^37^ and Principal stratum analysis on a population of interest defined by an intercurrent event (e.g. patients that would not discontinue treatment due to lack of efficacy) via the principal stratification method.^18^, ^38^, ^39^

### 2.4 Description and specification of the models

The models are described and specified below (Box 1 and Table 1) for two randomised treatments with six follow‐up visits. The ANCOVA estimator uses baseline and end of trial measurements, MMRM uses all repeated measurements.

**BOX 1:** Model descriptions

*ANCOVA following multiple imputation of missing MADRS10 outcomes* We used ANCOVA with multiple imputation for missing MADRS10 outcomes as follows^53^: For multiple imputation the predictors used were treatment, baseline MADRS10 outcome values and available outcomes measured at any post‐baseline visits. The method used is ‘predictive mean matching’^33^, ^53^ and we imputed *m* = 10 datasets. To each imputed dataset, we fitted an ANCOVA model. We modelled the MADRS10 score as a function of treatment and baseline MADRS10 outcome values to derive corresponding sets of statistics (treatment effect estimates and standard errors). We used Rubin's rules for imputation to combine the statistics.^54^
*Mixed‐models for repeated measurements* We modelled post‐baseline MADRS10 outcomes with a MMRM^34^ model allowing for different visit‐specific treatment effects (‘saturated’). We modelled the (variation of) repeated measurements by specifying random intercepts (between‐patient variation) and an unstructured serial correlation (within‐patient variation).
*Pattern‐mixture mixed models* We modelled a constant difference in treatment effect between patterns and same time profile in the placebo group for each pattern. The choice of patterns is based on timing of intercurrent events that caused monotone missing data. The estimated covariance matrix from the fitted model is used to estimate the weighted standard error for the weighted average . We used three different patterns described below to support the pattern mixture analyses. Monotone missing data refers to all outcome data being missing following an intercurrent event. Completers and quasicompleters: all outcomes available, or last visit outcome available with previous visits outcomes intermittently missing, or only last visit missing outcome with previous visits outcomes available fully or intermittently missing. Late visit dropout: last two visits with monotone missing outcomes, previous visits outcomes available fully or intermittently missing. Early visits dropout: monotone missing outcomes starting from week 2, week 3 or week 4 (missing thereon until the end of trial).
*MMRM following jump‐to‐reference (J2R) multiple imputation* A MMRM model is fitted on the reference arm (placebo) using only the baseline outcome values and time as fixed effects. Missing outcome values following intercurrent events for patients in the experimental arm are imputed in two steps. First, the reference arm model is used to predict the ‘fixed part’ of the imputed outcome from the baseline outcome and the time of missing value in order to match the patients on the reference arm from which outcomes will be used (‘jumped‐to’). To the predicted ‘fixed’ outcome values a random error is added to enable multiple imputation. The added random errors are drawn from the distributions of errors estimated from the MMRM model fit on the reference arm at each corresponding visit j). The final imputed outcome values were not rounded. We then complete the dataset with these imputed values for the experimental arm. Then, we fit a MMRM model on the imputed dataset (on all data, both experimental and reference arm patients) and follow the same steps as for ‘MMRM without imputation’ analysis method to derive the treatment effect estimate and the standard error.^51^ The intermittent and monotone missing outcomes in the control arm are considered MAR and are not imputed with the jump‐to‐reference approach. We imputed *m* = 10 datasets to which we fit the MMRM model to derive m corresponding sets of statistics (treatment effect estimates and standard errors). We used Rubin's rules for imputation to combine these statistics.^54^
*Principal stratum analysis on a population of interest defined by an intercurrent event (e.g. principal stratum of patients who would not discontinue treatment due to lack of efficacy) – compliers average causal effect (CACE) estimate with respect to the intercurrent event of interest (e.g. treatment discontinuation due to any reason)*. A logistic regression model is used to derive propensity scores for each patient (potential outcome) to experience the intercurrent event of interest. A cut‐off value is chosen to identify the patients that would not experience the intercurrent event (predicted principal stratum of compliers with regards to treatment discontinuation due to any reason). On this stratum of (imperfectly) identified patients (*not the same as the observed compliers or completers*), we apply the MMRM model as described above, without any imputation, and with the same model specification. The use of MMRM as an estimator was driven by the longitudinal design with repeated measures; other estimators could also be used, for example ANCOVA.

**TABLE 1:** Model specification and notation

Analysis method	Model	Notation
ANCOVA	$Y_{i}=\beta_{0}+\beta_{1}\times \text{Treatmen}t_{i}+\beta_{2}\times \text{BaselineValu}e_{i}+\epsilon_{i}$	$Y_{i}$ = MADRS10 outcome for patient i at planned end of trial visit at 6 weeks $\epsilon_{i}∼N\left(0,\sigma_{\epsilon_{i}}^{2}\right)$ $\text{Treatmen}t_{i}$ = randomised treatment for patient i, indicator is 0 for control and 1 for treatment Average difference in means (δ) between treatment and placebo at end of trial: $\delta =\beta_{1}$
MMRM	$Y_{\mathrm{ij}}=\beta_{0}+\beta_{1}\times \text{Treatmen}t_{i}+\beta_{2F}\times \mathrm{Tim}e_{\mathrm{ij}}^{\left(F\right)}+\beta_{3F}\times \mathrm{Tim}e_{\mathrm{ij}}^{\left(F\right)}\times \text{Treatmen}t_{i}+\beta_{4}\times \text{BaselineValu}e_{i}+b_{i}+\epsilon_{\mathrm{ij}}$	$Y_{\mathrm{ij}}$ = MADRS10 outcome for patient i at follow‐up visit j j = follow‐up visit 1 to 6 $\epsilon_{\mathrm{ij}}∼N\left(0,\sum_{\epsilon_{\mathrm{ij}}}^{2}\right)$ $b_{i}∼N\left(0,D\right)$ $\sum_{\epsilon_{\mathrm{ij}}}^{2}\;$= unstructured correlation matrix to model correlation of repeated measurements in time within patient $b_{i}$ = random intercepts for patients D = covariance matrix of random effects $b_{i}$ *b*_*i*_ and $\epsilon_{\mathrm{ij}}$ are assumed independent $\mathrm{Tim}e_{\mathrm{ij}}^{\left(F\right)}$ = time index for patient *i* being at follow‐up visit *F* ($\mathrm{Tim}e_{\mathrm{ij}}^{\left(F\right)}$ = 1 if *j = F*, $\mathrm{Tim}e_{\mathrm{ij}}^{\left(F\right)}$ = 0 if *j ≠ F*), *F* = 2,…, 6 $\beta_{1}$ = average difference in MADRS10 between treatment and placebo group at follow‐up visit 1 (treatment effect at follow‐up visit 1) $\beta_{0}=$ average MADRS10 score in placebo arm at follow‐up visit 1 (only post‐baseline outcomes are modelled) *β*_2*F*_ = average change in MADRS10 for placebo group from first follow‐up visit to follow‐up visit F *β*_3*F*_ = average difference in effect on MADRS10 between follow‐up visit F and follow‐up visit 1 *β*_4_ = average influence of baseline MADRS10 outcome values on the post‐baseline MADRS10 outcomes Average difference in means (δ) between treatment and placebo at visit 6 (at week 6): $\delta_{6}=\beta_{1}+\beta_{36}$
PMMM	$Y_{\mathrm{ij}}=\beta_{0}+\beta_{1}\times \text{Treatmen}t_{i}+\beta_{2F}\times \mathrm{Tim}e_{\mathrm{ij}}^{\left(F\right)}+\beta_{3F}\times \mathrm{Tim}e_{\mathrm{ij}}^{\left(F\right)}\times \text{Treatmen}t_{i}+\beta_{4}\times \text{BaselineValu}e_{i}+\beta_{5P}\times \text{Patter}n_{i}^{\left(P\right)}+\beta_{6P}\times {\text{Treatment}}_{i}\times \text{Patter}n_{i}^{\left(P\right)}+b_{i}+\epsilon_{\mathrm{ij}}$	$\beta_{2F}=$ average change in MADRS10 for placebo group from first follow‐up visit to follow‐up visit F $\beta_{3F}=$ average difference between treatment and placebo in the change in MADRS10 between follow‐up visit F and follow‐up visit 1 *β*_4_ = average influence of baseline MADRS10 score on the post‐baseline MADRS10 outcomes *P* = denotes the pattern for each patient, reference is pattern 1 (completers and quasicompleters) *β*_5*P*_ = average difference on MADRS10 in pattern *P* compared to pattern 1 (completers and quasicompleters) in placebo group at follow‐up visit 1 *β*_6*P*_ = average difference in effect on MADRS10 between pattern *P* and pattern 1 (completers and quasicompleters) Difference in means (δ) between treatment and placebo at visit 6 at week 6, taking into account the dropout patterns: $\delta_{6P}=\beta_{1}+\beta_{36}+\beta_{6P}$ And the estimated treatment effect from the PMMM is the average of these pattern‐specific effects weighted by their occurrence in the sample. The estimated covariance matrix from the fitted model is used to estimate the weighted standard error for the weighted average—Adjusted *δ*_6_.

## 3 RESULTS

### 3.1 Studies included

Of the six included studies, 003‐002, 003‐020, 003‐021 and 003‐022 were conducted in USA. Studies 84023 and 85027 were conducted in Finland and the United Kingdom, respectively. All studies were multicentre, with parallel groups, patients were randomised to placebo, mirtazapine or amitriptyline in 003‐020, 003‐021 and 003‐022, and to placebo or mirtazapine in 003‐002, 84023 and 85027. The sample sizes range between 90 and 150 randomised patients. All studies included adult patients suffering from depression. Study 84023 was an inpatient trial.^19^ Further information can be found in the Supporting Information S1.

### 3.2 Missing data and intercurrent events

Data were missing monotonously, intermittently, or both, with different patterns in each study (Figures 1, 2, 3, 4, and Supporting Information S2. Percentages of total missing outcome data at trial level ranged from 9.9% (003‐022) to 24.1% (003‐002 and 003‐021), with different distributions between arms and visits. There were mostly monotone missing data in study 84023 (21.3%) and 85027 (12.6%), and both monotone and intermittent missing data in the other studies. (See Figures 2 and 3 and Supporting Information S1.) Percentages of patients with missing outcomes at planned end of study visits differed within studies between treatment groups, and varied between studies, ranging from 16.3% (003‐022) to 40.9% (003‐021) in mirtazapine arms, from 19.7% (85027) to 56.8% (003‐002) in placebo arms, and from 18.4% (003‐022) to 31.9% (003‐021) in amitriptyline arms.

The type, occurrence, frequency and timing of treatment discontinuation varied between treatment groups within studies, and between studies. Treatment discontinuations were mainly related with AEs or LoE (Figure 4 for study 003‐002, Table 3 and Supporting Information S2 for the other five studies).

### 3.3 Estimands corresponding to analysis methods

We were able to map six different estimands to the common analyses investigated in this research (Table 2). The same estimand can be targeted by different analyses, differing through the assumptions made at statistical estimation level. In Table 2 we mention fully only the attributes that are different from those mentioned earlier in the table. If an attribute is the same as in the previously described estimand, it is not explicitly described again. Furthermore, in Table 2, in column ‘Description of the other estimand attribute(s) and formulated clinical questions’, we provide for all derived estimands the corresponding clinical questions.

**TABLE 2:** Analysis methods and corresponding derived estimands

No.	Method	Strategy/ies for intercurrent events	Description of the formulated clinical questions and other estimand attribute(s)	Necessary missing data assumptions for estimand
1	ANCOVA on complete cases	Patients with missing outcomes at end of trial because of treatment discontinuation at any timepoint due to AEs/LoE/other reasons are not included in the analysis. Hypothetical strategy for treatment discontinuation due to AEs/LoE/other reasons. Treatment policy strategy for other intercurrent events.	*What is the improvement achieved if no patients stopped the treatment?* *What is the treatment effect on MADRS10 in adults suffering from depression after 6 weeks* ^a^ *of treatment administered as the only medication to treat depression compared to no treatment being taken, had the treatment discontinuation due to AEs/LoE/other reasons not occurred, and if patients who discontinued were to have similar disease course afterwards as patients taking the treatment but did not discontinue, and regardless of other intercurrent events?* ***[Estimand 1 ANCOVA]***	MCAR for missing data at end of trial for an unbiased estimate.^b^ Assumptions: patients who discontinued treatment due to AEs/LoE/other reasons constitute a random sample of all included patients. No assumption needed for intermittent missing outcomes.
		Patients with missing outcomes at end of trial because of treatment discontinuation at any timepoint due to AEs/LoE/other reasons are not of interest, and are not included in the analysis. Treatment policy strategy for other intercurrent events.	*What is the improvement achieved in those patients that can complete the treatment?* Population: Adults suffering from depression who complete the study and the treatment. ***This is not a population that can be defined outside the clinical trial*.** Population‐level summary: Difference between experimental treatment completers and control^c^ treatment completers in mean MADRS10 score after 6 weeks^a^ of treatment **There is no estimand corresponding to this commonly employed analysis.** Or this is a principal stratum estimand, but the estimator is biased.^55^	If estimation is aimed at the difference between completers in the experimental arm and completers from the control arm, then no assumption is made for missing data.
2	ANCOVA following LOCF imputation	While on treatment strategy for treatment discontinuation at any timepoint due to AEs/LoE/other reasons. Treatment policy strategy for intercurrent events that lead to intermittent missing outcomes or do not cause missing outcomes but may impact the efficacy estimate.	*What is the improvement achieved before treatment had to be discontinued?* Population‐level summary: Difference between experimental treatment and control^c^ in mean MADRS10 score **prior to treatment discontinuation** within a maximum of 6 weeks^a^ of treatment *What is the treatment effect on MADRS10 in adults suffering from depression prior to treatment discontinuation due to AEs/LoE/other reasons, treatment being administered as the only medication to treat depression, compared to no treatment being taken and regardless of other intercurrent events?* ***[Estimand 2 ANCOVA]***	There is no need for additional missing data assumptions. Values following treatment discontinuation are not of interest for the research question. There is no fixed point in time for the contrast, hence no condition to estimate it at a later (or earlier) timepoint for which there would be the need to make assumptions about missing data.
		Treatment policy strategy for treatment discontinuation at any timepoint due to AEs/LoE/other reasons. Treatment policy strategy for the other intercurrent events.	*What is the improvement achieved regardless of any treatment stopping?* *What is the treatment effect on MADRS10 in adults suffering from depression after 6 weeks* ^a^ *of treatment administered as the only medication to treat depression compared to no treatment being taken, regardless of any intercurrent events?* ***[Estimand 3 ANCOVA]***	For an unbiased estimate^b^ the distribution of the observed outcomes at end of trial and LOCF imputed outcomes at end of trial must be the same as the distribution of the observed and unobserved outcomes at end of trial. Assumptions for imputation: patients who discontinued treatment due to AEs/LoE/other reasons were not to deteriorate or improve their disease course afterwards. No assumption needed for intermittent missing outcomes.
3	MMRM following LOCF imputation	Treatment policy strategy for treatment discontinuation at any timepoint due to AEs/LoE/other reasons. Treatment policy strategy for the other intercurrent events.	*What is the improvement achieved regardless of any treatment stopping?* *What is the treatment effect on MADRS10 in adults suffering from depression after 6 weeks* ^a^ *of treatment administered as the only medication to treat depression compared to no treatment being taken, regardless of any intercurrent events?* ***[Estimand 3 MMRM]***	For an unbiased estimate^b^ the distribution of the observed outcomes and imputed outcomes (LOCF) must be the same as the distribution of the observed and unobserved outcomes. Imputed outcomes are the last available outcome values for patients with monotone missing outcomes and are immediately previous available outcome values for patients with intermittent missing outcomes. Assumptions: patients who discontinued treatment due to AEs/LoE/other reasons were not to deteriorate or improve their disease course afterwards.
4	ANCOVA following multiple imputation	Hypothetical strategy for treatment discontinuation at any timepoint due to AEs/LoE/other reasons. Assumptions: adults suffering from depression who discontinued treatment due to AEs/LoE/other reasons were to have similar disease course afterwards as similar patients taking the treatment but did not experience it. Treatment policy strategy for the other intercurrent events.	*What is the improvement achieved if no patients stopped the treatment?* *What is the treatment effect on MADRS10 in adults suffering from depression after 6 weeks* ^a^ *of treatment administered as the only medication to treat depression compared to no treatment being taken, had the treatment discontinuation due to AEs/LoE/other reasons not occurred?* ***[Estimand 1 ANCOVA]***	MAR for missing data imputation for an unbiased estimate.^b^ Assumptions: patients who discontinued treatment due to AEs/LoE/other reasons were to have similar disease course afterwards as similar patients (based on covariates in the model) who did not discontinue.
		Treatment policy strategy for treatment discontinuation at any timepoint due to AEs/LoE/other reasons. Treatment policy strategy for the other intercurrent events.	*What is the improvement achieved regardless of any treatment stopping?* *What is the treatment effect on MADRS10 in adults suffering from depression after 6 weeks* ^a^ *of treatment administered as the only medication to treat depression compared to no treatment being taken, regardless of any intercurrent events?* ***[Estimand 3 ANCOVA]***	MAR for missing data imputation for an unbiased estimate.^b^
5	MMRM without imputation	Hypothetical strategy for treatment interruption or discontinuation at any timepoint due to AEs/LoE/other reasons. Assumptions: adults suffering from depression who discontinued treatment due to AEs/LoE/other reasons were to have similar disease course afterwards as similar patients taking the treatment but did not experience it. Treatment policy strategy for the other intercurrent events.	*What is the improvement achieved if no patients stopped the treatment?* *What is the treatment effect on MADRS10 in adults suffering from depression after 6 weeks* ^a^ *of treatment administered as the only medication to treat depression compared to no treatment being taken, had the treatment discontinuation due to AEs/LoE/other reasons not occurred?* ***[Estimand 1 MMRM]***	MAR for intermittent and monotone missing data for an unbiased estimate.^b^
		Treatment policy strategy for treatment discontinuation at any timepoint due to AEs/LoE/other reasons. Treatment policy strategy for the other intercurrent events.	*What is the improvement achieved regardless of any treatment stopping?* *What is the treatment effect on MADRS10 in adults suffering from depression after 6 weeks* ^a^ *of treatment administered as the only medication to treat depression compared to no treatment being taken, regardless of any intercurrent events?* ***[Estimand 3 MMRM]***	MAR for intermittent and monotone missing data for an unbiased estimate.^b^
6	MMRM following J2R imputation	A composite strategy for treatment discontinuation at any timepoint due to AEs/LoE/other reasons. Assumptions: adults suffering from depression who discontinued treatment due to AEs/LoE/other reasons were to have similar disease course afterwards as similar patients that were taking other treatment and did not discontinue treatment. Treatment policy strategy for the other intercurrent events. **(!) This interpretation may not correspond to a clinically meaningful question.**	*What is the improvement achieved when some patients stop treatment, but clinical outcome is assigned to no improvement after stopping treatment?* Variable: composite of all measured MADRS10 outcomes for patients who do not discontinue treatment, and, for patients who discontinue: of measured MADRS10 before discontinuation, and of assigned MADRS10 values after discontinuation, that were from similar patients from the reference arm that did not discontinue. *What is the treatment effect on MADRS10 in adults suffering from depression after 6 weeks* ^a^ *of treatment administered as the only medication to treat depression compared to no treatment being taken, in terms of the composite variable, and regardless of other intercurrent events?* ***[Estimand 4 MMRM]***	For control (reference) arm: MAR for intermittent and monotone missing data for an unbiased estimate.^b^ MAR for intermittent missing data.
		Treatment policy strategy for treatment discontinuation at any timepoint due to AEs/LoE/other reasons. Treatment policy strategy for the other intercurrent events.	*What is the improvement achieved regardless of any treatment stopping?* *What is the treatment effect on MADRS10 in adults suffering from depression after 6 weeks* ^a^ *of treatment administered as the only medication to treat depression compared to no treatment being taken, regardless of any intercurrent events?* ***[Estimand 3 MMRM]***	For control (reference) arm: MAR for intermittent and monotone missing data for an unbiased estimate.^b^ For the experimental arm: MNAR for monotone missing data where missing intervention data is considered to jump to reference. MAR for intermittent missing data.
7	Pattern‐mixture mixed model (PMMM) Patterns based on treatment discontinuation times and type of missing data	Composite strategy 1 for early treatment discontinuation (from week 2, 3 or 4) due to AEs/LoE/other reasons. Composite strategy 2 for late treatment discontinuation (from week 5) due to AEs/LoE/other reasons. Assumptions for composite strategies are different based on timing of treatment discontinuation. Assumptions: adults suffering from depression who discontinued treatment due to AEs/LoE/other reasons were to have afterwards the same disease course as observed before the treatment discontinuation according to the pattern where they belong to. Treatment policy strategy for the other intercurrent events. **(!) This interpretation may not correspond to a clinically meaningful question.**	*What is the improvement achieved when some patients stop treatment, but improvement is assigned to continue unchanged after stopping treatment?* Variable: composite of all measured MADRS10 outcomes for patients who do not discontinue treatment, and, for patients who discontinue, of measured MADRS10 before discontinuation and of assigned MADRS10 values after discontinuation, that were extrapolated from the treatment effect achieved before discontinuation, differently for early or late discontinuation for patients who discontinue. *What is the treatment effect on MADRS10 in adults suffering from depression after 6 weeks* ^a^ *of treatment administered as the only medication to treat depression compared to no treatment being taken, in terms of the composite variable, and regardless of other intercurrent events?* ***[Estimand 5 MMRM]***	MAR for intermittent missing data.
		Treatment policy strategy for treatment discontinuation due to AEs/LoE/other reasons. Treatment policy strategy for the other intercurrent events.	*What is the improvement achieved regardless of any treatment stopping?* *What is the treatment effect on MADRS10 in adults suffering from depression after 6 weeks* ^a^ *of treatment administered as the only medication to treat depression compared to no treatment being taken, regardless of any intercurrent events? **[Estimand 3 MMRM]***	MNAR for monotone missing data with missing data dependent on the patterns (of missing data). MAR for intermittent missing data.
8	Principal stratum analysis	Principal stratum strategy for a specific intercurrent event of interest (or multiple intercurrent events aggregated) such as treatment discontinuation at any timepoint due to e.g., AEs). Hypothetical strategy for treatment discontinuation due to any other reason than the one of interest, e.g., due to LoE. Treatment policy for the other intercurrent events.	*What is the improvement achieved in those patients that can/could complete the treatment?* Population: Adults suffering from depression that would not discontinue treatment due to AEs/LoE/other reasons; patients that would not discontinue treatment due to the intercurrent event of interest *What is the treatment effect on MADRS10 in adults suffering from depression after 6 weeks* ^a^ *of treatment administered as the only medication to treat depression compared to no treatment being taken, that would not experience the intercurrent event of interest, and regardless of other intercurrent events?* ***[Estimand 6 MMRM]***	Data missing in a population of interest is MNAR. The population of interest that did not have missing data in the experimental arm would not have had missing data in the control arm either. Data missing due to other reasons than in the population of interest is MAR.

*Abbreviations*: AEs, adverse events; LoE, lack of efficacy; MCAR, missing completely at random; MAR, missing at random; MNAR, missing not at random.

^a^ End of trial is at 6 weeks for studies 003‐002, 84023 and 003‐020, 003‐021 and 003‐022, and at 5 weeks for study 85027 (see Figure 1).

^b^ Unbiased estimate refers to the estimate if the outcomes would have been observed fully (no missing outcomes).

^c^ Experimental treatment is mirtazapine in all studies, control is placebo in studies 003‐002, 84023 and 85027, control is placebo and amitriptyline is active control in studies 003‐020, 003‐021 and 003‐022.

**TABLE 3:** Intercurrent events (treatment discontinuations due to different reasons)

	Intercurrent events *n* (%)											
Study	Arm	AE	AE and LoE	Drug unrelated	LoE	Unk	Efficacy	Insufficient compliance	Insufficient compliance and LoE	AE and insufficient compliance	AE and insufficient compliance and LoE	Insufficient compliance and drug unrelated
	003‐002 (*N* = 88)											
	Placebo	0(0)	2(2.3)	2(2.3)	18(20.5)	3(3.4)	0	0	0	0	0	0
	Mirtazapine	4(4.5)	0(0)	2(2.3)	8(9.1)	3(3.4)	0	0	0	0	0	0
	84023 (*N* = 105)											
	Placebo	0(0)	0	4(3.8)	14(13.4)	1(1.0)	0(0)	2(1.9)	1(1.0)	0	0	0
	Mirtazapine	1(1.0)	0	2(1.9)	10(9.5)	3(2.9)	2(1.9)	1(1.0)	1(1.0)	0	0	0
	85027 (*N* = 124)											
	Placebo	1(0.8)	0	0(0)	7(5.6)	2(1.6)	0(0)	2(1.6)	0	0(0)	0(0)	1(0.8)
	Mirtazapine	3(2.4)	0	1(0.8)	6(4.8)	3(2.4)	1(0.8)	0(0)	0	1(0.8)	1(0.8)	0(0)
	003‐020 (*N* = 114)											
	Amitriptyline	4(3.5)	4(3.5)	3(2.6)	0(0)	0(0)	1(0.9)	0	0	0	0	0
	Placebo	2(1.8)	1(0.9)	4(3.5)	3(2.6)	2(1.8)	0(0)	0	0	0	0	0
	Mirtazapine	2(1.8)	2(1.8)	5(4.4)	2(1.8)	2(1.8)	1(0.9)	0	0	0	0	0
	003‐021 (*N* = 139)											
	Amitriptyline	5(3.6)	3(2.2)	3(2.2)	0(0)	4(2.9)	0	0	0	0	0	0
	Placebo	1(0.7)	2(1.4)	6(4.3)	15(10.8)	4(2.9)	0	0	0	0	0	0
	Mirtazapine	1(0.7)	0(0.0)	3(2.2)	13(9.4)	2(1.4)	0	0	0	0	0	0
	003‐022 (*N* = 148)											
	Amitriptyline	3(2.0)	1(0.7)	2(1.4)	3(2.0)	0(0)	0(0)	0	0	0	0	0
	Placebo	0(0)	2(1.4)	3(2.0)	8(5.4)	0(0)	1(0.7)	0	0	0	0	0
	Mirtazapine	3(2.0)	0(0)	1(0.7)	3(2.0)	1(0.7)	0(0)	0	0	0	0	0

The treatment is the same for all estimands (Experimental treatment or control^c^ administered as the only medication to treat depression for 6 weeks^a^—see Figure 1). The target population is the same for all estimands (Adults suffering from depression), except for the estimand involving a principal stratum strategy (Adults suffering from depression that would not experience the intercurrent event of treatment discontinuation due to any reason). The variable of interest is the same for all estimands (MADRS10) (See Table 2). The population‐level summary is the same for all estimands (Difference between experimental treatment and control in mean MADRS10 score after 6 weeks^a^ of treatment, and in view of the variable definition), except for the estimand involving a while‐on‐treatment strategy (Difference between experimental treatment and control in mean MADRS10 score prior to treatment discontinuation within a maximum of 6 weeks^a^ of treatment). Hence, the major differences concern the different strategies for addressing intercurrent events of interest) Table 3.

ANCOVA on complete cases can only be mapped to an estimand after 6 weeks^a^ of treatment for the target population under the assumption that completers are a random sample from all patients included in the study. If this were to hold, it would constitute a treatment policy strategy for the intercurrent event.

For analyses that use LOCF imputation (ANCOVA and MMRM), we were able to formulate two estimands that capture the treatment effect in the target population. One estimand could define a treatment effect with a treatment policy strategy for intercurrent events under the assumption that patients' outcomes remain unchanged after their last observation before stopping treatment. This is not hypothetical in the sense of ‘the intercurrent event would not occur’. The second estimand strategy for intercurrent events we identified for ANCOVA following LOCF imputation is while‐on‐treatment: the last available assessment is analysed as last value on treatment (what the treatment was able to achieve before it was stopped), and not defined in terms of time since start of treatment.

ANCOVA following multiple imputation and MMRM without imputation essentially target the same estimands under similar assumptions, only following different analysis strategies. They lead to estimands prespecified with a single hypothetical strategy for addressing all intercurrent events, and could only be considered as targeting an estimand with a single treatment policy strategy for addressing all intercurrent events, if the ‘missing at random’ assumption holds for the occurrence of intercurrent events.

MMRM following J2R imputation and PMMM may aim at an estimand prespecified with a treatment policy strategy for addressing all intercurrent events. They may also aim at a composite variable strategy to ensure an appropriate outcome after the intercurrent event occurred can be included. When the treatment policy strategy is intended, MMRM following J2R imputation makes the strong assumption that following treatment discontinuation, the patient will not take any other treatment than the reference (placebo in these trials). However, depending on the reference treatment this situation might rarely lead to a relevant estimand, unless the reference treatment would be the usual treatment to switch to in case of treatment failure.

In the principal stratum analysis, an attempt is made for an estimand prespecified with a principal stratum strategy for the intercurrent event of interest (and hypothetical strategy for other intercurrent events).

All analyses involving imputation could also be interpreted as trying to estimate a treatment effect having observed all outcomes at end of treatment period, hence, an estimand prespecified with treatment policy as single strategy for addressing all intercurrent events. In absence of actually observed outcomes, an imputation of the missing outcomes completes the dataset and artificially allows a treatment policy strategy. This approach can only lead to a viable estimand prespecified with a single treatment policy strategy for all intercurrent events, if the model and the strong assumptions for imputation match a realistic scenario of the changes to outcomes after the intercurrent event.

### 3.4 Observed differences in effect estimates

**FIGURE 5:**
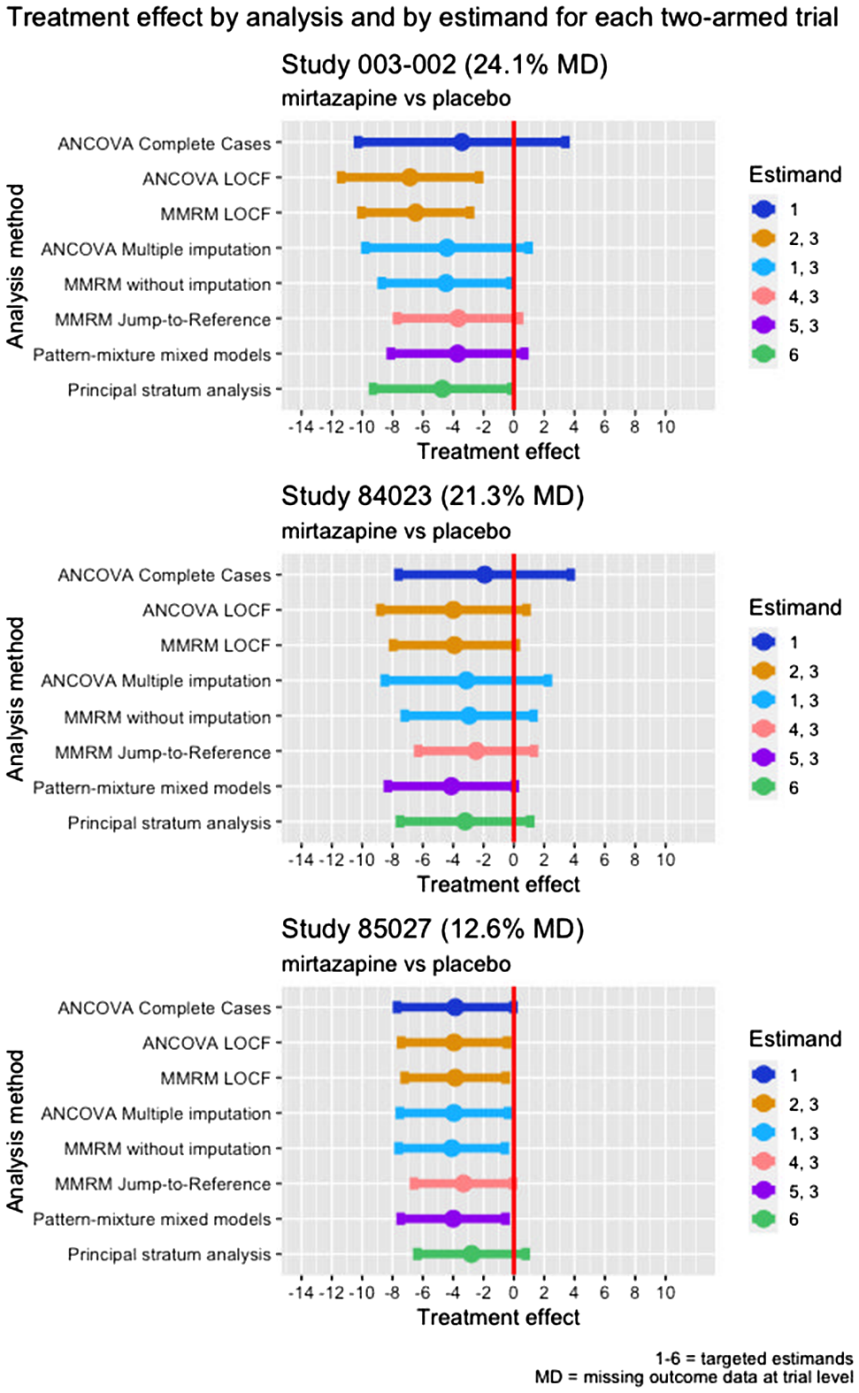
Forest plot treatment effects studies 003‐002, 84023 and 85027

**FIGURE 6:**
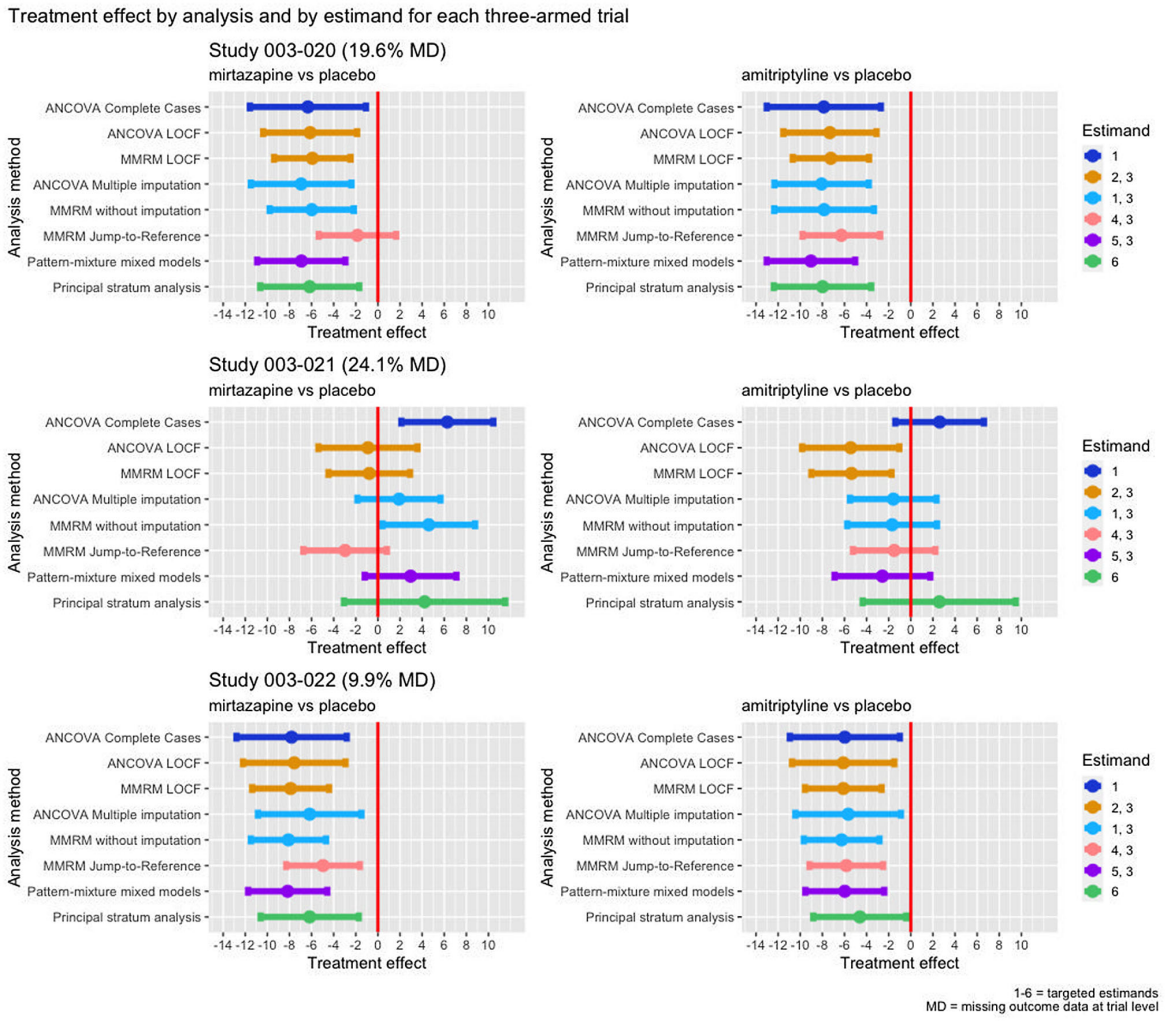
Forest plot treatment effects studies 003‐020, 003‐021 and 003‐022

Re‐analysis of the six trials using the methods described above largely yielded comparable direction of estimated treatment effects across studies. The range of point estimates was of 4–8 points average reduction in MADRS10 in favour of mirtazapine and amitriptyline compared to placebo (Figures 5 and 6, and Supporting Information S3. A clear exception was study 003‐021, showing larger differences between analyses (direction and size) and also differences compared to other studies. Additionally, some analyses (ANCOVA on complete cases, MMRM following J2R and PMMM in 003‐002; ANCOVA on complete cases, ANCOVA following multiple imputation, MMRM following J2R and principal stratum analysis in 84023; MMRM following J2R in 003‐020) deviated from the general correspondence of effect estimates.

Studies 85027 and 003‐022 recorded the smallest percentages of treatment discontinuations (12.6% and 9.9%). In 85027, they were mostly due to LoE, balanced between arms. In 003‐022, they were mostly due to AEs in amitriptyline and mirtazapine arms, and LoE in placebo arm. There were small or no amounts of intermittent missing data. The direction and size of treatment effects are similar across analyses, principal stratum analysis in study 85027 being slightly different.

Study 003‐020 recorded a considerable amount of treatment discontinuations (29.2%), mostly due to AEs or AEs and LoE, and relatively more frequently in the amitriptyline arm; comparable amount of intermittent missing data as study 003‐022. For study 003‐020 the direction and size of treatment effects are comparable across analyses, except those yielded by MMRM following jump‐to‐reference imputation, which are prominently smaller for mirtazapine when compared to placebo. Jump‐to‐reference imputation assumes that patients who discontinue treatment in the experimental arm continue afterwards with an outcome trajectory as if they did not take treatment (in trial as if randomised to placebo) from treatment discontinuation onwards.

Studies 003‐002 and 84023 recorded large amounts of treatment discontinuations (46% and 36%), especially due to LoE in both studies and both arms, with no intermittent missing data in study 84023 and with a large amount of intermittent missing data in study 003‐002. Direction and size of treatment effects are comparable across all analyses except those involving LOCF, which showed larger estimates.

Study 003‐021 also recorded a large amount of treatment discontinuations (41.3%), especially due to LoE in placebo and mirtazapine arms and especially due to AEs in the amitriptyline arm. Direction and size of treatment effects are heterogeneous across analyses. The completers from the placebo arm recorded better outcomes than both amitriptyline and mirtazapine arms completers, hence the seemingly negative effect suggested by the ANCOVA conducted on complete cases. However, this analysis ignores the different reasons and mechanisms leading to treatment discontinuations between arms. Also, this analysis shows results similar to the principal stratum analysis. The analysis involving jump‐to‐reference imputation provides contrasting results as treatment discontinuations from treatment arms will be imputed with outcomes from the placebo arm, and since the placebo arm recorded more improvement than the treatment arms, this analysis provides the opposing direction of treatment effects. Although across most studies results were similar, this study demonstrates a larger impact of the choice of estimand.

Overall, MMRM without imputation yielded similar treatment effect estimates as PMMM. When applying PMMM, first an imputation under MNAR is done, and after this step a MMRM analysis is conducted. PMMM seems to result in slightly larger treatment effects in some cases and this could be due to the fact that for each treatment discontinuation pattern, the treatment effect (slope per pattern) is considered to be retained to the end of the trial.

ANCOVA following multiple imputation yields similar treatment effects with MMRM without imputation, with wider confidence intervals generated by ANCOVA as it uses less data (baseline and endpoint outcomes). Supporting Information S3 contains the table with treatment effects and 95% CI reflected in Figures 5 and 6.

### 3.5 Sensitivity analyses

We found different estimators (analyses) targeting the same estimand. When targeting the same estimand, described by the same attributes, these analyses can be sensitivity analysis for each other, provided the assumptions are different and these differences are specified explicitly. For example, when targeting estimand 1 (Table 2), MMRM (MAR) can be a sensitivity analysis for ANCOVA on complete cases (MCAR), but MMRM (MAR) cannot be a sensitivity analysis for ANCOVA following multiple imputation (MAR). When compared to other analyses, if a single attribute is different in an estimand derived from an analysis, then the targeted estimand is different. Consequently, if the targeted estimand is different, that particular analysis cannot be a sensitivity analysis for the other analyses against which is compared. For example, the difference between MMRM following J2R when targeting estimand 5 and PMMM when targeting estimand 6 is at the level of the variable attribute. Hence, they cannot be used as sensitivity analysis.

In our research it is clear that the same derived estimand could correspond to different analyses (e.g. estimand 4 targeted by ANCOVA following LOCF, MMRM following LOCF, ANCOVA following multiple imputation, MMRM, MMRM following J2R and PMMM). Conversely, the same analysis could correspond to different estimands (e.g. PMMM possibly targeting estimand 6 and estimand 4). The choice of analysis intended for sensitivity analysis purposes must match the estimand targeted by the primary analysis.

## 4 DISCUSSION

Investigating the analysis methods using the estimands framework demanded to investigate the estimand attributes and the assumptions made at the level of each analysis method at a fine granularity of detail. Estimands highlight there are different intercurrent events (e.g. study discontinuation due to AEs or LoE, instead of generic ‘missing data’), different strategies for intercurrent events and different assumptions made by each analysis. For most of re‐analysed trials we found quantitatively comparable numerical results, although some were notably different, such as most analyses in study 003‐021 or MMRM following J2R in study 003‐020. These showed a different size of treatment effect or both a different size and direction of treatment effect when compared to the other studies. These differences were driven by type (mostly LoE), frequency (large amounts of discontinuations due to LoE) and timing of intercurrent events occurrence (early and/or late in the trial).

This mapping exercise was not simple and did not lead to uniquely defined estimands: the same statistical analysis could be matched to more than one estimand; the reciprocal is also true. This suggests the need for pre‐specification at trial design stage and benefit from the E9(R1) estimands framework as the analysis alone does not clarify what is being estimated.

ANCOVA on complete cases does not unconditionally aim at a meaningful target population, hence may not provide a useful estimand. If the completers represent a random sample from all patients included in the trial, it can be argued whether the treatment policy estimand is estimated. However, because of the strong assumptions needed, the value of this type of analysis is questionable when used in evaluating clinical trials efficacy.

The only interpretable strategy for intercurrent events we identified for analyses involving LOCF, was for ANCOVA following LOCF imputation, and is the while‐on‐treatment strategy, as in this case study discontinuation equals treatment discontinuation. It is difficult to say in general if a while‐on‐treatment strategy is relevant or not; it may be of limited value for depression trials of short duration. We could map an estimand with a treatment policy strategy to ANCOVA following LOCF and MMRM following LOCF. However, we think that LOCF imputation was often not applied for a treatment policy strategy per se, but rather to have an evaluable outcome at the end of the trial such that ITT principle could be applied.

We interpreted the composite variable strategy as prespecifying an outcome value to be incorporated in the variable, to reflect the intercurrent event. One well‐known application of the composite variable strategy is, for instance, non‐responder imputation. If a patient experiences an intercurrent event or withdraws from the study for any reason, then that patient is assigned a value to reflect ‘non‐response’, specifically to reflect the intercurrent event as treatment failure. This could be a value ‘0’ to reflect the intercurrent event as non‐response (e.g. if outcome is dichotomous).

Another interpretation of the composite variable strategy could be when using a composite outcome, such as progression‐free survival (PFS) for instance. Other interpretations of the composite strategy are also possible. The ICH E9(R1) does not restrict the use to only binary outcomes and suggests that it can be on a continuous scale too. Furthermore, the framework states that ‘An intercurrent event is considered in itself to be informative about the patient's outcome and is therefore incorporated into the definition of the variable’. This leaves other options open, and it does not state precisely whether the outcome to be assigned that reflects the intercurrent event should be fixed or stochastic, single general value for all patients or subject‐specific. In this research, we interpreted the composite variable strategy also as prespecifying an outcome value that is non‐fixed. Hence, we considered a jump‐to‐reference imputation and PMMM a composite variable strategy, as the value assigned is non‐constant over patients (subject‐specific), and it reflects the intercurrent event (e.g. treatment discontinuation).

With some creativity, another strategy for addressing intercurrent events that can be derived from ANCOVA following LOCF and MMRM following LOCF could alternatively be a ‘composite variable’ strategy: for patients that withdrew from the study, this intercurrent event is incorporated in the variable definition by using the patients' last measured outcome. The Addendum provides a definition that is larger than the definition of a classical categorical composite outcome. The authors' interpretation (for ANCOVA following LOCF) is that it cannot be only one direction (‘bad’/’good’). With LOCF, the clinical meaning can go both ways and is difficult to be clinically interpreted; this duplicate meaning is complicated for a composite variable strategy and also depends on the disease (LOCF in Alzheimer's disease vs LOCF in depression). How the intercurrent events are incorporated in the variable definition does matter in order to have an interpretable estimand. Some ways the composite variable strategy is implemented can be of more interest and more plausible than others.

For MMRM without imputation the point estimates and direction were close across studies with those from MMRM following J2R imputation and PMMM, and slightly closer to PMMM results. In the re‐analysed six trials, for some of them the treatment effects were small and overall there are differences to be observed—but in most cases the 95% CI were largely overlapping. The observed results in this research suggest the MAR assumption can be robust to deviations, although this is not generally supported.^40^, ^41^, ^42^ MMRM could be considered a reliable and relatively simple starting point as primary efficacy analysis for short‐term depression trials.

MMRM following J2R imputation can target a treatment policy treatment effect at the end of trial (6 weeks^a^) where outcomes after treatment discontinuation are supposed to follow a stochastic trajectory as if they received reference (placebo in our studies). This assumption needs careful consideration depending on availability of alternative treatments or standard of care in order to avoid over‐conservativeness or misinterpretation of treatment effects. Furthermore, depending on the percentages of treatment discontinuations at trial level and on how imbalanced they are between arms, the estimation following J2R imputation aiming at a treatment policy treatment effect at the end of trial can be severely biased. In other disease settings, the ‘reference’ or the arm which patients ‘jump’ to may still be a realistic situation. For instance, if the reference is another treatment or standard of care well defined and standardised, and if the ‘jump’ is part of the standard of care policy.

PMMM can target an estimand prespecified with a single treatment policy strategy for addressing all intercurrent events, where the outcome after treatment discontinuation is assumed to follow a stochastic trajectory conditional on the timing of discontinuation. This assumption is difficult to justify from a clinical viewpoint or verify because very few data are collected after treatment discontinuation. Indeed, the addendum suggests timing can be a differentiating factor. For each of these two intercurrent events a different composite variable strategy can be selected. Depending on the patient behaviour, in other settings more than two intercurrent events could be defined. Consequently, the pattern‐mixture used in the analysis should be adapted accordingly.

Although it may be debatable and have some limitations, the J2R and pattern‐mixture could correspond to clinically relevant questions.

The principal stratum analysis targets a treatment effect in a stratum of patients that likely will not discontinue treatment. This treatment effect is of great interest. However, it can be a difficult estimand to estimate, because for instance, collected covariates may not be strong predictors of the intercurrent event or there are only few intercurrent events of interest. Although principal stratum strategy is one of the five strategies defined by the Addendum, it is not a commonly employed analysis used in estimation of treatment effects in depression. It could be relevant, and it was used in this paper to illustrate how the strategy could be applied and how the treatment effect in such a principal stratum can be estimated.

Lastly, occurrence of missing data is often encountered in clinical trials, especially in longitudinal studies with multiple planned visits for outcome measurement.^29^, ^43^ ‘Missing data’ is a multi‐dimensional concept. There is a vast amount of methods developed to deal with missing covariates or missing outcomes.^36^, ^37^, ^44^, ^45^, ^46^, ^47^, ^48^, ^49^, ^50^, ^51^, ^52^ The amount of missing data should not replace clinical rationale as the driver for the constructed target estimand. Trialists and stakeholders should define the primary objective and the corresponding estimand when designing the trial. Given the estimand and thus the strategies for addressing intercurrent events, missing data should be as much as possible limited by design, data collection and the analysis choice. Data that are nevertheless missing at trial completion are then a problem of estimation and not a problem of targeted estimand. It is up to thorough debate and further evaluation (e.g. with sensitivity analyses) whether the estimator of the targeted estimand is biased and how biased it is and how reliant it is on modelling assumptions regarding missing data not addressed in the estimand.

Our research has several strengths: we systematically translated a range of established analysis methods into estimands according to the E9(R1) Addendum, with a full description of its attributes and the clinical question of interest. We provide in‐depth and transparency‐enhancing details regarding embedded and implicit decisions, and assumptions made, at analysis or imputation level for each method. We acknowledge the estimands are not free of assumptions, but being more explicit about the assumptions made, will facilitate the understanding and interpretability of estimands.

Our research has limitations: This research does not start from formulating de novo meaningful research questions and these trials did not have a priori pre‐specified estimands as per E9(R1). Derived estimands are for trials conducted before the estimands framework and we have to make do with available collected data long before the estimands framework. For instance, we do not have data for ‘other reasons for treatment discontinuation’ and we ‘constructed’ for illustrative purposes a shared intercurrent event for all analyses (any treatment discontinuation, without differentiating by reason where possible), of which we are fully aware it is not fully clinically justified. However, we think this research will improve understanding of the methods used, and improve comparison of analysis on trials that have been initiated before and after introduction of the estimands framework.

Results and conclusions may not be necessarily generalisable to other disease settings or to other type of trials or designs of trials. We conducted the analyses precisely as they are currently being performed, without making differentiation between reasons for treatment discontinuation. It is anticipated that with differentiation in handling different intercurrent events with different strategies, the analyses will possibly yield different results to some extent. However, the possible expected impact would be small for this particular disease and setting; even with (further) differentiation of strategies by intercurrent events, as AEs occurred early in the trials, LoE later or spread‐out throughout the entire trial duration, but there were mostly discontinuations due to LoE.

In most cases, there are two or three different estimands mapped for each analysis method. In absence of a precise clinical question articulated beforehand, more than one interpretation of strategies for intercurrent events could be derived, hence more than one estimand. It is therefore not possible to indicate which of the possibly targeted estimands was the actual intended one. This uncertainty does not exist if we methodically move from estimands through design and analysis. In order to avoid ambiguity regarding targeted estimands, it is paramount to start trial planning from the clinical question to be answered linked with the objective of the trial. This question should be precisely mirrored in the estimand attributes, before deciding which is the suitable estimator for it.

## 5 CONCLUSION

In the re‐analysed trials, the quantitative differences between the population‐level summaries of these estimands were overall small, so in this particular example there was limited impact on the clinical interpretation of the trial results. Not all six estimands had a clinically meaningful interpretation. Only a few analyses would target the same estimand, hence by definition few could be used as sensitivity analyses. The fact that an analysis could estimate different estimands emphasises the importance of prospectively defining the estimands targeting the primary objective of a trial. The fact that an estimand can be targeted by different analyses emphasises the importance of prespecifying precisely the estimator for the targeted estimand.

## AUTHOR CONTRIBUTIONS

Marian Mitroiu produced and refined several drafts and iterations of this manuscript following thorough input from Steven Teerenstra, Katrien Oude Rengerink, Frank Pétavy and Kit C. B. Roes. All authors critically revised and approved the final version of this manuscript.

## CONFLICT OF INTEREST

The authors declare that there is no conflict of interest.

## Supporting information


**Appendix S1** Supporting Information

## Data Availability

Data were provided by MSD for research purposes only, with ownership and authority to share remaining at MSD.
