# Supplementary material for: Estimation of treatment effects in short‐term depression studies. An evaluation based on the ICH E9(R1) estimands framework
Source: Pharm Stat. 2022 Jun 9;21(5):1037–57. doi: 10.1002/pst.2214 (PMC9543408; doi:10.1002/pst.2214)
Supplement: Supplementary file 1 — Appendix S1 Supporting Information [file PST-21-1037-s001.zip › PST_2214_pst-21-0016-File008.pdf]

Estimation of treatment effects in longitudinal depression studies. An application of ICH E9(R1) estimands framework

Supplementary material

April 2020

Descriptive statistics of studies characteristics

1. Study 003-002

**Treatment group/N Patients**	Randomised	ITT
Mirtazapine	45	44
Placebo	45	44

Visit	collected outcomes (n)	mean MADRS10	sd MADRS10	missing outcomes (ITT-n)	missing %
Mirtazapine					
1	42	21.6	8.1	2	4.5
2	41	16.3	9.1	3	6.8
3	36	16.0	8.3	8	18.2
4	36	15.6	9.8	8	18.2
5	33	13.1	10.1	11	25.0
6	28	12.3	10.6	16	36.4
Placebo					
1	42	28.2	5.3	2	4.5
2	37	25.8	7.9	7	15.9
3	34	23.9	9.2	10	22.7
4	31	22.4	10.8	13	29.5
5	22	18.1	11.5	22	50.0
6	19	15.9	12.0	25	56.8

Clinical outcome parameters based on ITT (All randomised patients with at least 1 post-baseline outcome value available)

Follow-up: Visit number coincides with week number

2. Study 84023

**Treatment group/N Patients**	Randomised	ITT
Mirtazapine	59	54
Placebo	55	51

Visit	collected outcomes (n)	mean MADRS10	sd MADRS10	missing outcomes (ITT-n)	missing %
Mirtazapine					
1	54	24.9	8.4	0	0.0
2	40	18.1	9.5	14	25.9
3	34	15.2	11.6	20	37.0
Placebo					
1	51	26.4	10.4	0	0.0
2	40	21.6	9.2	11	21.6
3	29	17.4	10.7	22	43.1

Clinical outcome parameters based on ITT (All randomised patients with at least 1 post-baseline outcome value available)

Follow-up: Visit 1 is at week 2, Visit 2 is at week 4, Visit 3 is at week 6

3. Study 85027

**Treatment group/N Patients**	Randomised	ITT
Mirtazapine	66	63
Placebo	66	61

Visit	collected outcomes (n)	mean MADRS10	sd MADRS10	missing outcomes (ITT-n)	missing %
Mirtazapine					
1	63	20.0	9.4	0	0.0
2	51	12.9	9.2	12	19.0
3	48	11.9	9.5	15	23.8
Placebo					
1	60	20.4	9.3	1	1.6
2	54	16.9	9.8	7	11.5
3	49	14.7	10.3	12	19.7

Clinical outcome parameters based on ITT (All randomised patients with at least 1 post-baseline outcome value available)

Follow-up: Visit 1 is at week 2, Visit 2 is at week 4, Visit 3 is at week 5

4. Study 003-020

**Treatment group/N Patients**	Randomised	ITT
Amitriptyline	43	38
Mirtazapine	44	39
Placebo	43	37

Visit	collected outcomes (n)	mean MADRS10	sd MADRS10	missing outcomes (ITT-n)	missing %
Amitriptyline					
1	37	25.1	6.7	1	2.6
2	36	20.6	7.8	2	5.3
3	32	19.9	8.3	6	15.8
4	31	16.6	8.5	7	18.4
5	25	15.6	9.1	13	34.2
6	26	13.5	9.5	12	31.6
Mirtazapine					
1	39	23.6	6.9	0	0.0
2	36	21.6	7.4	3	7.7
3	33	18.9	9.4	6	15.4
4	25	17.7	8.4	14	35.9
5	24	16.4	7.4	15	38.5
6	25	14.1	8.1	14	35.9
Placebo					
1	37	26.6	9.9	0	0.0
2	31	25.7	10.5	6	16.2
3	31	25.7	12.1	6	16.2
4	29	23.4	10.9	8	21.6
5	28	20.6	13.3	9	24.3
6	25	21.6	11.7	12	32.4

Clinical outcome parameters based on ITT

Follow-up: Visit number coincides with week number

5. Study 003-021

**Treatment group/N Patients**	Randomised	ITT
Amitriptyline	50	47
Mirtazapine	50	44
Placebo	50	48

Visit	collected outcomes (n)	mean MADRS10	sd MADRS10	missing outcomes (ITT-n)	missing %
Amitriptyline					
1	45	21.6	7.6	2	4.3
2	41	20.0	7.2	6	12.8
3	34	16.0	8.1	13	27.7
4	36	12.5	7.5	11	23.4
5	31	12.5	8.3	16	34.0
6	32	10.5	7.4	15	31.9
Mirtazapine					
1	42	23.0	7.3	2	4.5
2	40	19.0	9.6	4	9.1
3	39	18.7	10.6	5	11.4
4	39	16.9	11.4	5	11.4
5	26	12.6	8.0	18	40.9
6	26	13.7	8.1	18	40.9
Placebo					
1	48	26.8	7.6	0	0.0
2	42	23.1	9.1	6	12.5
3	36	19.7	10.4	12	25.0
4	32	20.3	10.7	16	33.3
5	23	15.5	10.5	25	52.1
6	21	7.5	5.2	27	56.2

Clinical outcome parameters based on ITT (All randomised patients with at least 1 post-baseline outcome value available)

Follow-up: Visit number coincides with week number

6. Study 003-022

**Treatment group/N Patients**	Randomised	ITT
Amitriptyline	50	49
Mirtazapine	50	49
Placebo	50	50

Visit	collected outcomes (n)	mean MADRS10	sd MADRS10	missing outcomes (ITT-n)	missing %
Amitriptyline					
1	49	32.6	6.2	0	0.0
2	47	26.6	6.8	2	4.1
3	45	22.4	7.7	4	8.2
4	42	20.5	9.2	7	14.3
5	40	17.7	9.7	9	18.4
6	40	18.0	10.3	9	18.4
Mirtazapine					
1	49	33.5	6.4	0	0.0
2	47	28.9	7.4	2	4.1
3	46	27.0	7.7	3	6.1
4	43	23.2	8.6	6	12.2
5	42	19.9	9.9	7	14.3
6	41	16.5	12.0	8	16.3
Placebo					
1	50	34.0	5.1	0	0.0
2	48	31.0	5.8	2	4.0
3	46	27.9	8.1	4	8.0
4	45	25.2	9.6	5	10.0
5	42	23.7	10.1	8	16.0
6	38	23.9	10.9	12	24.0

Clinical outcome parameters based on ITT (All randomised patients with at least 1 post-baseline outcome value available)

Follow-up: Visit number coincides with week number
