# Supplementary material for: Estimation of treatment effects in short‐term depression studies. An evaluation based on the ICH E9(R1) estimands framework
Source: Pharm Stat. 2022 Jun 9;21(5):1037–57. doi: 10.1002/pst.2214 (PMC9543408; doi:10.1002/pst.2214)
Supplement: Supplementary file 1 — Appendix S1 Supporting Information [file PST-21-1037-s001.zip › PST_2214_pst-21-0016-File010.docx]

Treatment effect results and its corresponding estimand, and by study and analysis method

| **Study ID** | **No.** | **Analysis and imputation method** | **Number of patients** | **Point estimate**  **for mean difference between mirtazapine and placebo.*** | **95% Confidence interval for treatment effect estimate** |
| --- | --- | --- | --- | --- | --- |
| **003-002** | **Timing of outcome data collection (6 time points) following randomisation:** Weeks: 1, 2, 3, 4, 5 and **6** | |  |  |  |
|  | **1** | **ANCOVA on complete cases** | N1=28  N0=19 | -3.43 | -10.27, 3.40 |
|  | **2** | **ANCOVA following LOCF imputation** | N1=44  N0=44 | -6.85 | -11.39, -2.29 |
|  | **3** | **MMRM following LOCF imputation** | N1=44  N0=44 | -6.47 | -10.05, -2.89 |
|  | **4** | **ANCOVA following multiple imputation** | N1=44  N0=44 | -4.41 | -9.79, 0.97 |
|  | **5** | **MMRM without imputation** | N1=44  N0=44 | -4.48 | - 8.71, -0.24 |
|  | **6** | **MMRM following J2R imputation** | N1=44  N0=44 | -3.68 | -7.70, 0.34 |
|  | **7** | **PMMM: patterns based on drop out times and type of missing data** | N1=44  N0=44 | -3.71 | -8.11, 0.69 |
|  | **8a** | **Propensity score for LoE discontinuations** | N1=#  N0=# | # | # |
|  | **8b** | **Propensity score for AE discontinuations** | N1=#  N0=# | # | # |
|  | **8c** | **Propensity score for ALL discontinuations** | N1=39  N0=35 | -4.72 | -9.28, -0.16 |
| **84023** | **Timing of outcome data collection (3 time points) following randomisation:** Weeks: 2, 4 and **6** | |  |  |  |
|  | **1** | **ANCOVA on complete cases** | N1=34  N0=29 | -1.93 | -7.61, 3.75 |
|  | **2** | **ANCOVA following LOCF imputation** | N1=54  N0=51 | -3.99 | -8.80, 0.83 |
|  | **3** | **MMRM following LOCF imputation** | N1=54  N0=51 | -3.92 | -7.96, 0.12 |
|  | **4** | **ANCOVA following multiple imputation** | N1=54  N0=51 | -3.14 | -8.51, 2.24 |
|  | **5** | **MMRM without imputation** | N1=54  N0=51 | -2.95 | -7.19, 1.29 |
|  | **6** | **MMRM following J2R imputation** | N1=54  N0=51 | -2.49 | -6.30, 1.32 |
|  | **7** | **PMMM: patterns based on drop out times and type of missing data** | N1=54  N0=51 | -4.12 | -8.30, 0.06 |
|  | **8a** | **Propensity score for LoE discontinuations** | N1=#  N0=# | # | # |
|  | **8b** | **Propensity score for AE discontinuations** | N1=#  N0=# | # | # |
|  | **8c** | **Propensity score for ALL discontinuations** | N1=53  N0=50 | -3.21 | -7.51, 1.08 |
| **85027** | **Timing of outcome data collection (3 time points) following randomisation:** Weeks: 2, 4 and **5** | |  |  |  |
|  | **1** | **ANCOVA on complete cases** | N1=48  N0=49 | -3.86 | -7.70, -0.03 |
|  | **2** | **ANCOVA following LOCF imputation** | N1=63  N0=61 | -3.94 | -7.43, -0.45 |
|  | **3** | **MMRM following LOCF imputation** | N1=63  N0=61 | -3.86 | -7.20, -0.53 |
|  | **4** | **ANCOVA following multiple imputation** | N1=63  N0=61 | -3.95 | -7.52, -0.39 |
|  | **5** | **MMRM without imputation** | N1=63  N0=61 | -4.09 | -7.60, -0.59 |
|  | **6** | **MMRM following J2R imputation** | N1=63  N0=61 | -3.32 | -6.58, -0.06 |
|  | **7** | **PMMM: patterns based on drop out times and type of missing data** | N1=63  N0=61 | -4.00 | -7.46, -0.55 |
|  | **8a** | **Propensity score for LoE discontinuations** | N1=#  N0=# | # | # |
|  | **8b** | **Propensity score for AE discontinuations** | N1=#  N0=# | # | # |
|  | **8c** | **Propensity score for ALL discontinuations** | N1=60  N0=51 | -2.79 | -6.36, 0.78 |
| **003-020** | **Timing of outcome data collection (6 time points) following randomisation: Weeks: 1, 2, 3, 4, 5 and 6** | |  |  |  |
|  | **1** | **ANCOVA on complete cases** | N1=25  N7=26  N0=25 | -6.33  -7.87 | -11.58, -1.08  -13.03, -2.71 |
|  | **2** | **ANCOVA following LOCF imputation** | N1=39  N7=38  N0=37 | -6.13  -7.32 | -10.38, -1.88  -11.53, -3.10 |
|  | **3** | **MMRM following LOCF imputation** | N1=39  N7=38  N0=37 | -5.93  -7.23 | -9.39, -2.47  -10.68, -3.78 |
|  | **4** | **ANCOVA following multiple imputation** | N1=39  N7=38  N0=37 | -6.94  -8.07 | -11.49, -2.39  -12.33, -3.80 |
|  | **5** | **MMRM without imputation** | N1=39  N7=38  N0=37 | -5.98  -7.84 | -9.79, -2.18  -12.35, -3.33 |
|  | **6** | **MMRM following J2R imputation** | N1=39  N7=38  N0=37 | -1.85  -6.28 | -5.35, 1.67  -9.79, -2.77 |
|  | **7** | **PMMM: patterns based on drop out times and type of missing data** | N1=39  N7=38  N0=37 | -6.92  -9.03 | -10.92, -2.94  -13.03, -5.03 |
|  | **8a** | **Propensity score for LoE discontinuations** | N1=#  N7=#  N0=# | #  # | #  # |
|  | **8b** | **Propensity score for AE discontinuations** | N1=#  N7=#  N0=# | #  # | #  # |
|  | **8c** | **Propensity score for ALL discontinuations** | N1=30  N7=30  N0=30 | -6.17  -7.98 | -10.65, -1.69  -12.39, -3.58 |
| **003-021** | **Timing of outcome data collection (6 time points) following randomisation: Weeks: 1, 2, 3, 4, 5 and 6** | |  |  |  |
|  | **1** | **ANCOVA on complete cases** | N1=26  N7=32  N0=21 | 6.28  2.61  TCPF7        -7.871659 | 2.12, 10.44  -1.40, 6.62 |
|  | **2** | **ANCOVA following LOCF imputation** | N1=44  N7=47  N0=48 | -0.90  -5.43 | -5.38, 3.58  -9.83, -1.04 |
|  | **3** | **MMRM following LOCF imputation** | N1=44  N7=47  N0=48 | -0.78  -5.36 | -4.47, 2.91  -8.99, -1.74 |
|  | **4** | **ANCOVA following multiple imputation** | N1=44  N7=47  N0=48 | 1.91  -1.58 | -1.85, 5.66  -5.50, 2.33 |
|  | **5** | **MMRM without imputation** | N1=44  N7=47  N0=48 | 4.60  -1.69 | 0.41, 8.79  -5.75, 2.37 |
|  | **6** | **MMRM following J2R imputation** | N1=44  N7=47  N0=48 | -2.97  -1.50 | -6.74, 0.81  -5.23, 2.22 |
|  | **7** | **PMMM: patterns based on drop out times and type of missing data** | N1=44  N7=47  N0=48 | 2.96  -2.57 | -1.19, 7.10  -6.89, 1.76 |
|  | **8a** | **Propensity score for LoE discontinuations** | N1=#  N7=#  N0=# | #  # | #  # |
|  | **8b** | **Propensity score for AE discontinuations** | N1=#  N7=#  N0=# | #  # | #  # |
|  | **8c** | **Propensity score for ALL discontinuations** | N1=9  N7=14  N0=10 | 4.22  2.58 | -3.07, 11.52  -4.35, 9.50 |
| **003-022** | **Timing of outcome data collection (6 time points) following randomisation: Weeks: 1, 2, 3, 4, 5 and 6** | |  |  |  |
|  | **1** | **ANCOVA on complete cases** | N1=41  N7=40  N0=38 | -7.81  -5.97  TCPF7        -7.871659 | -12.79, -2.82  -10.94, -0.99 |
|  | **2** | **ANCOVA following LOCF imputation** | N1=49  N7=49  N0=50 | -7.57  -6.11 | -12.20, -2.93  -10.74, -1.49 |
|  | **3** | **MMRM following LOCF imputation** | N1=49  N7=49  N0=50 | -7.89  -6.10 | -11.37, -4.42  -9.57, -2.64 |
|  | **4** | **ANCOVA following multiple imputation** | N7=49  N1=49  N0=50 | -6.17  -5.66 | -10.85, -1.49  -10.44, -0.89 |
|  | **5** | **MMRM without imputation** | N1=49  N7=49  N0=50 | -8.09  -6.26 | -11.49, -4,69  -9.68, -2.83 |
|  | **6** | **MMRM following J2R imputation** | N1=49  N7=49  N0=50 | -4.96  -5.83 | -8.29, -1.63  -9.16, -2.50 |
|  | **7** | **PMMM: patterns based on drop out times and type of missing data** | N1=49  N7=49  N0=50 | -8.16  -5.97 | -11.74, -4.58  -9.54, -2.40 |
|  | **8a** | **Propensity score for LoE discontinuations** | N1=#  N7=#  N0=# | #  # | #  # |
|  | **8b** | **Propensity score for AE discontinuations** | N1=#  N7=#  N0=# | #  # | #  # |
|  | **8c** | **Propensity score for ALL discontinuations** | N1=34  N7=40  N0=34 | -6.17  -4.61 | -10.61, -1.73  -8.82, -0.41 |

#The stratum of interest could not be identified based on available measured covariates

*For studies 003-020, 003-021 and 003-022 the treatment differences are between mirtazapine and placebo (above), and between amitriptyline and placebo (below)

**end of trial is at six weeks for studies 003-002, 84023 and 003-020, 003-021 and 003-022, and at five weeks for study 85027

N7= amitriptyline, N1= mirtazapine, N0= placebo
