# Supplementary figures and images for: Estimation of treatment effects in short‐term depression studies. An evaluation based on the ICH E9(R1) estimands framework
Source: Pharm Stat. 2022 Jun 9;21(5):1037–57. doi: 10.1002/pst.2214 (PMC9543408; doi:10.1002/pst.2214)

Supplemental material for heatmaps and individual trajectories for studies 84023, 85027, 003-020, 003-021 and 003-022


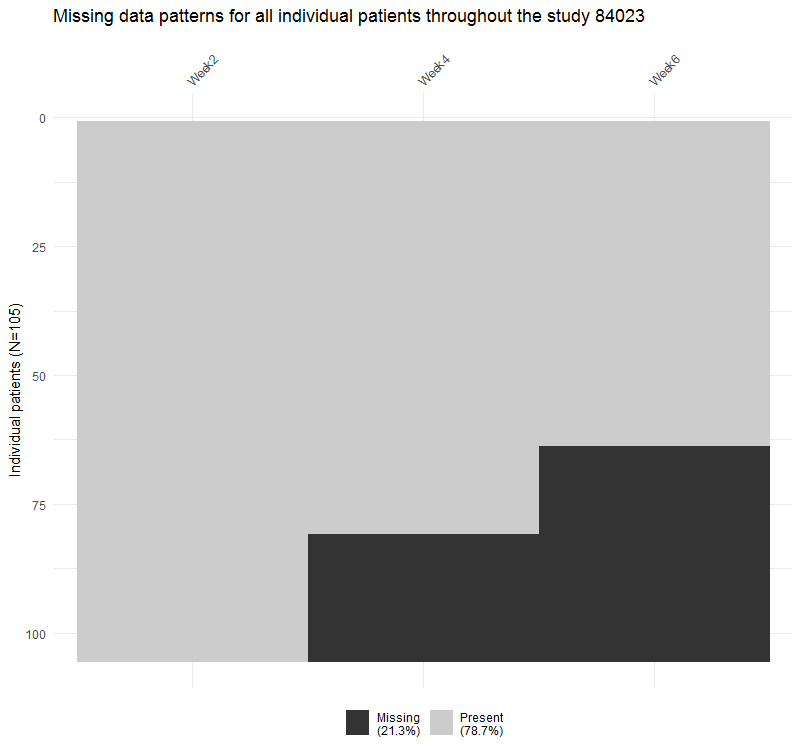


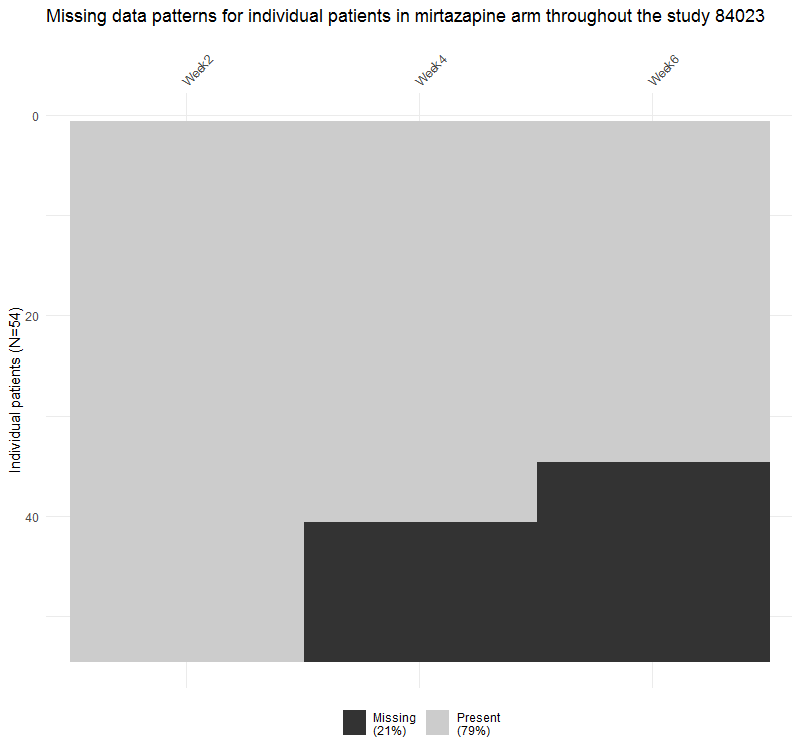

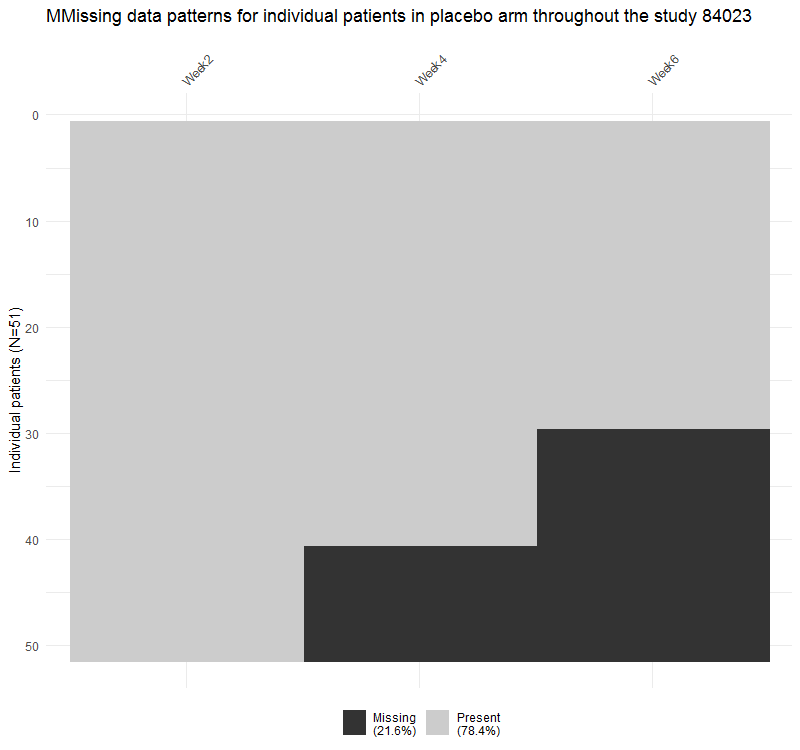


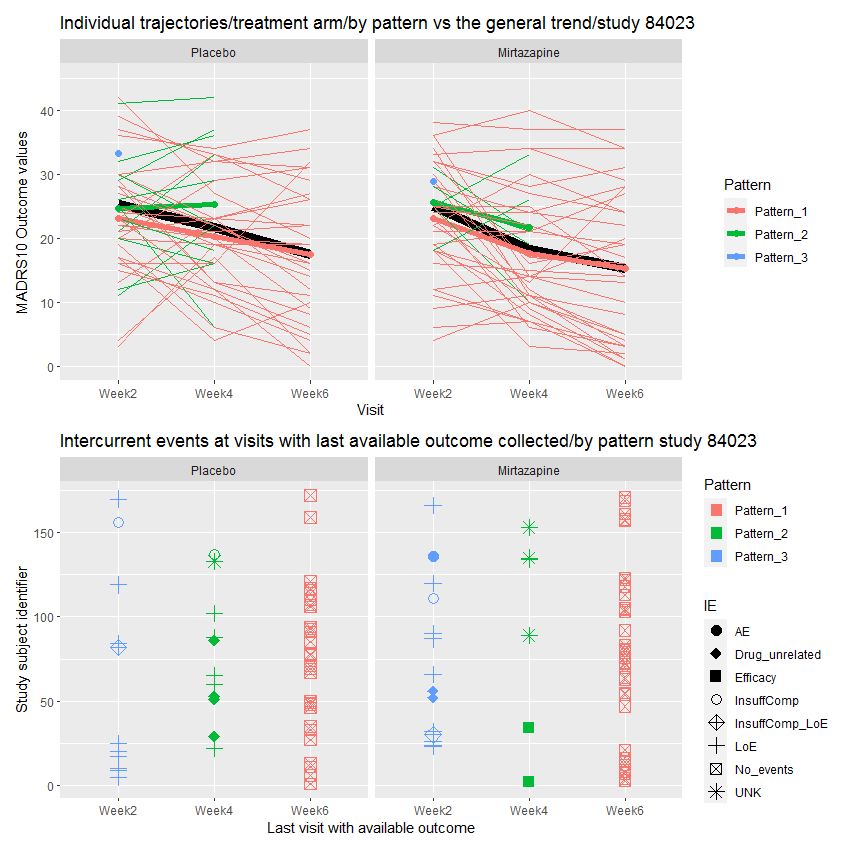


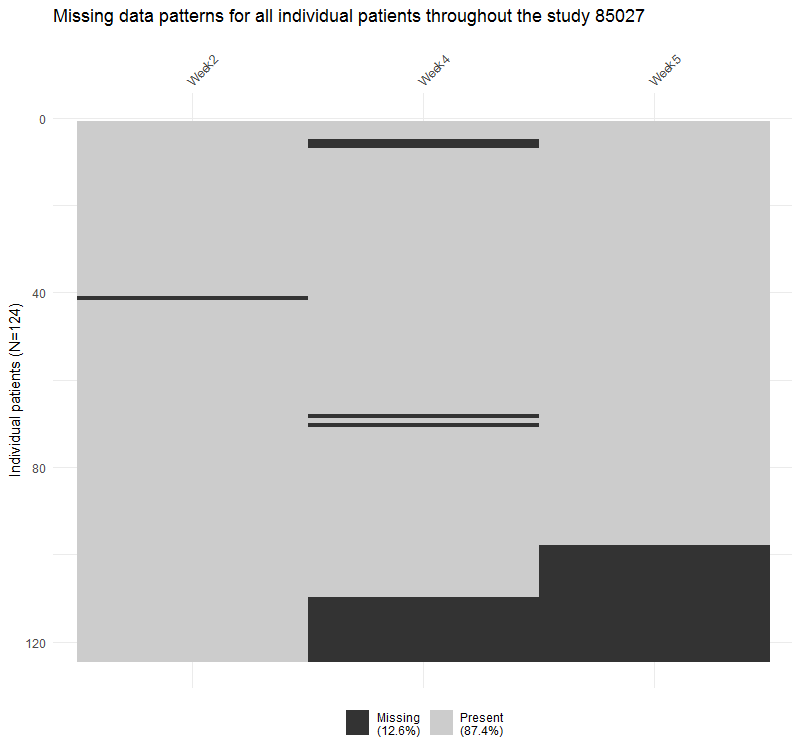

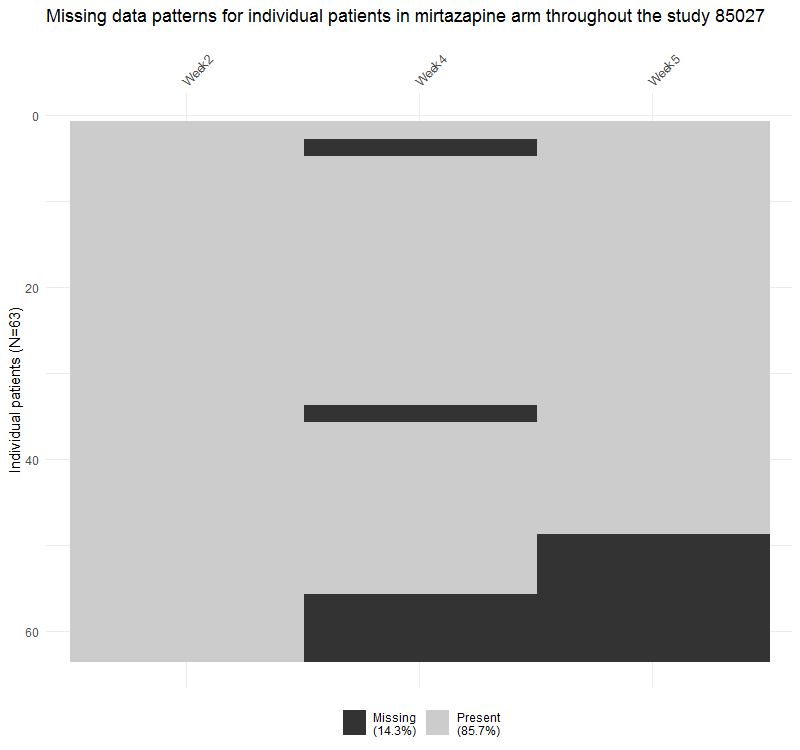

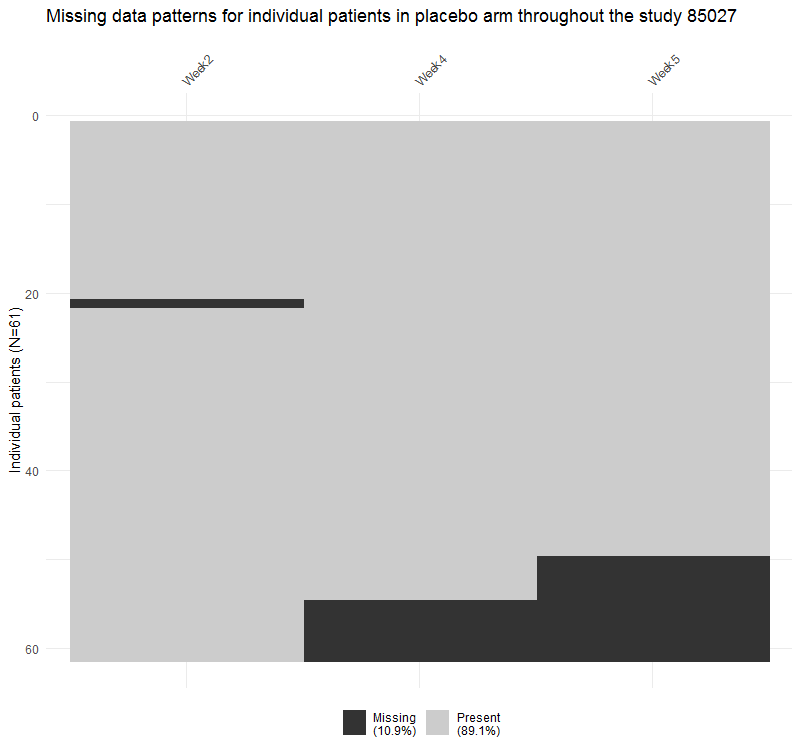


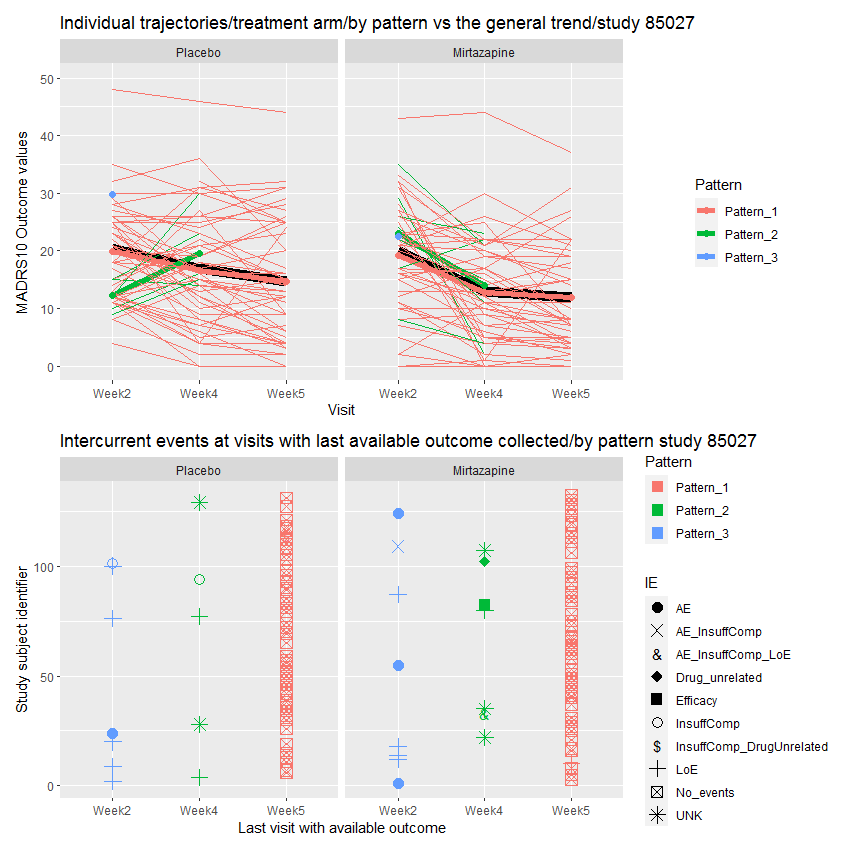


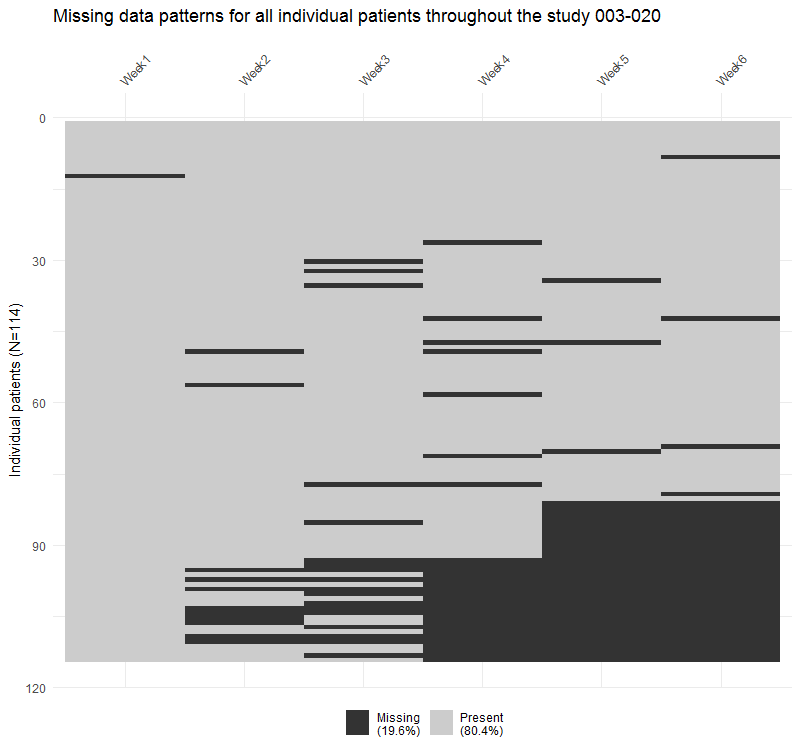


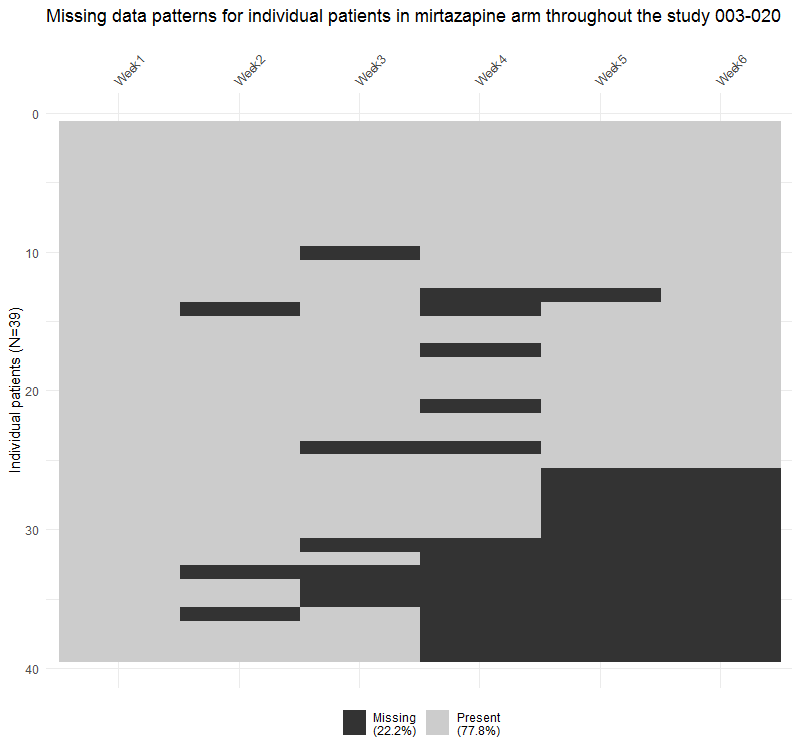

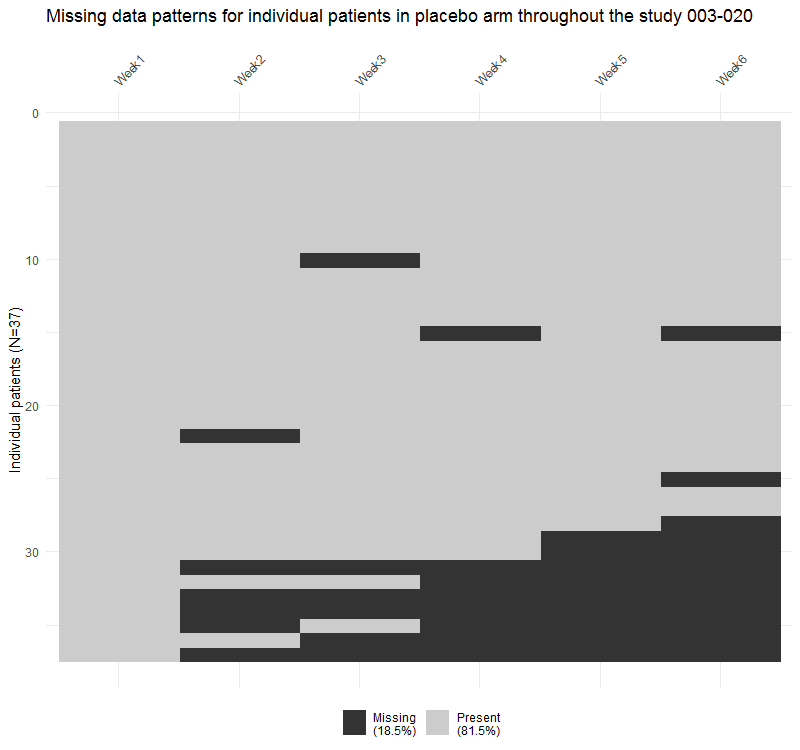

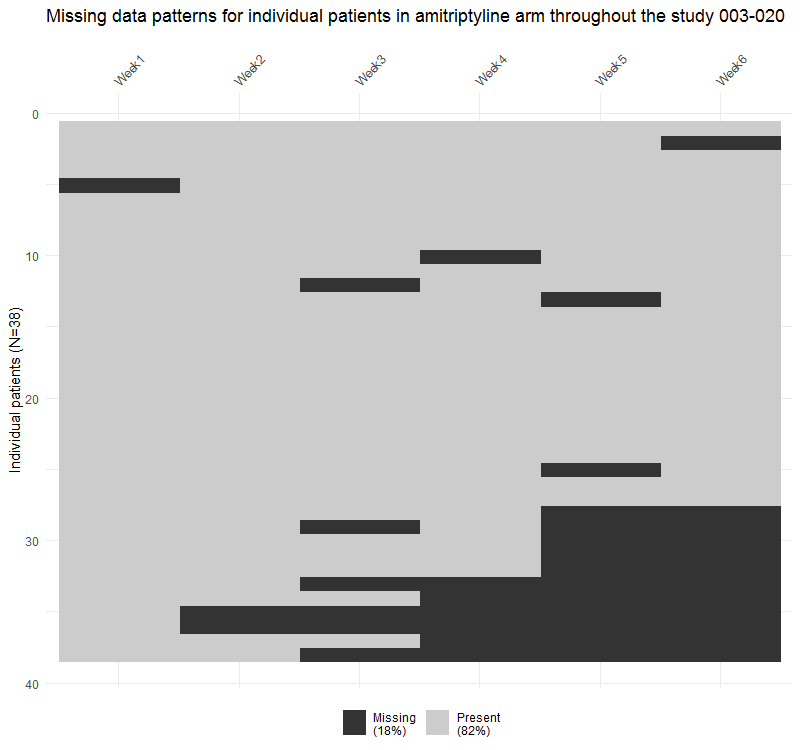


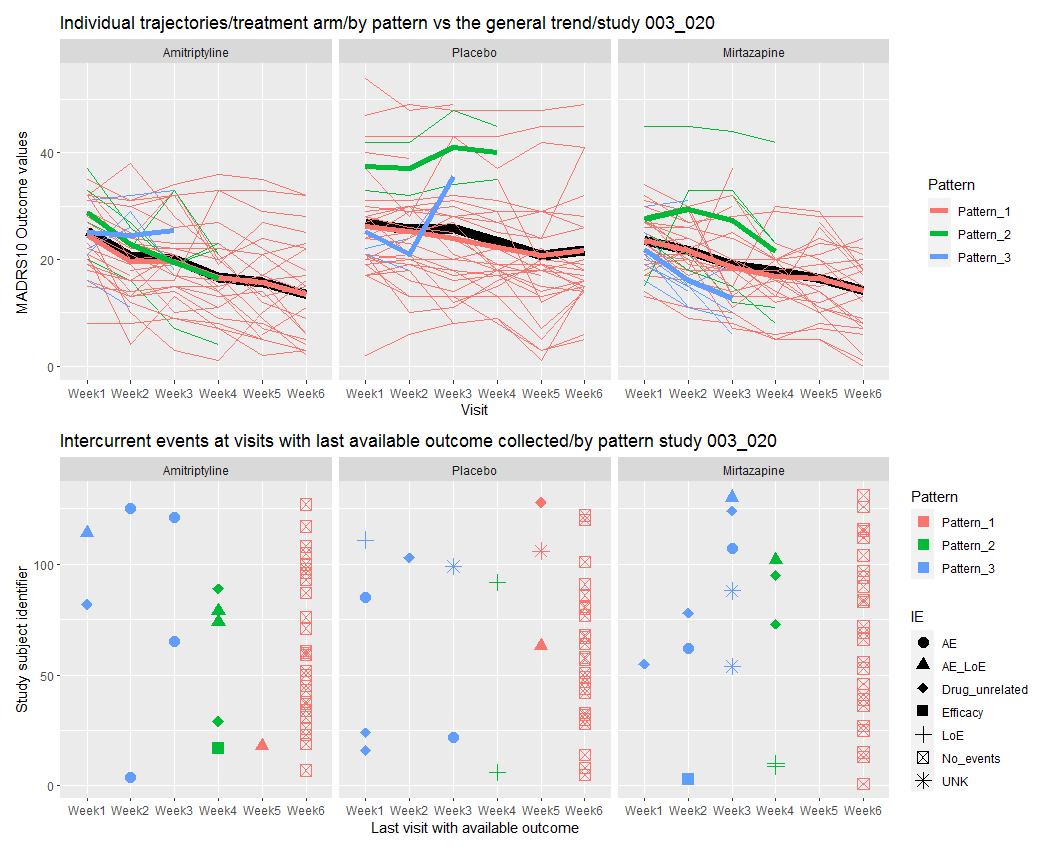


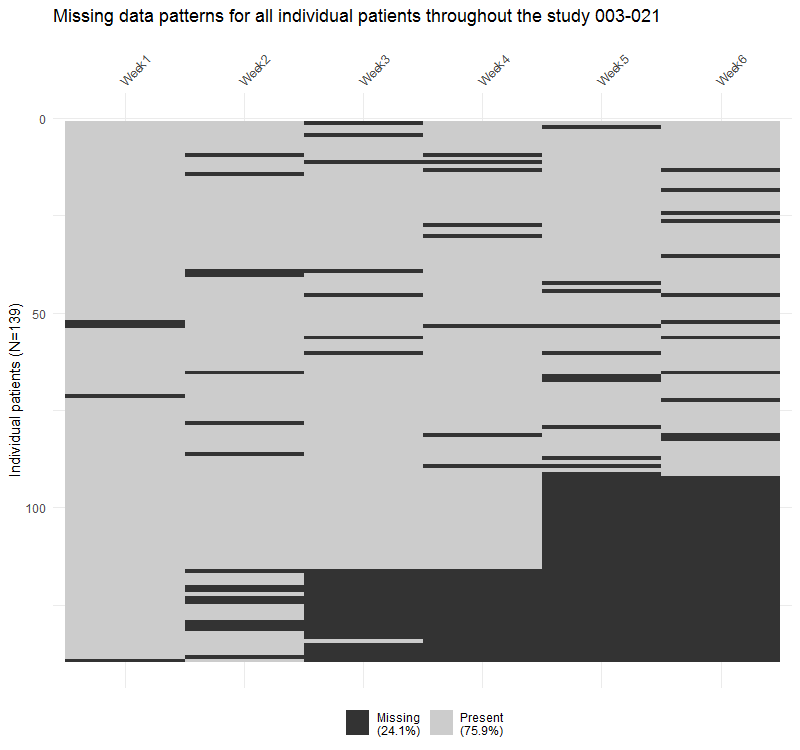


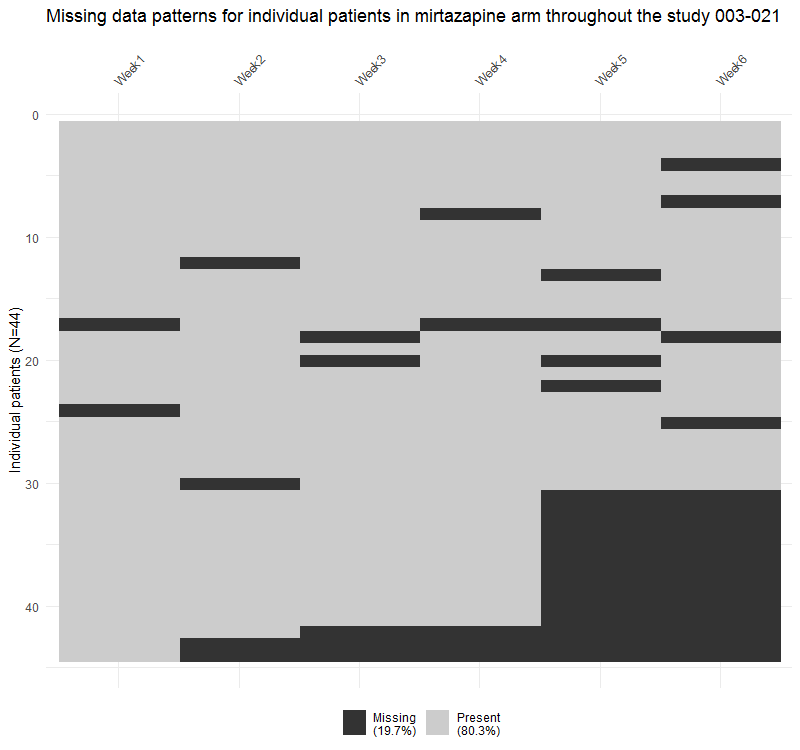

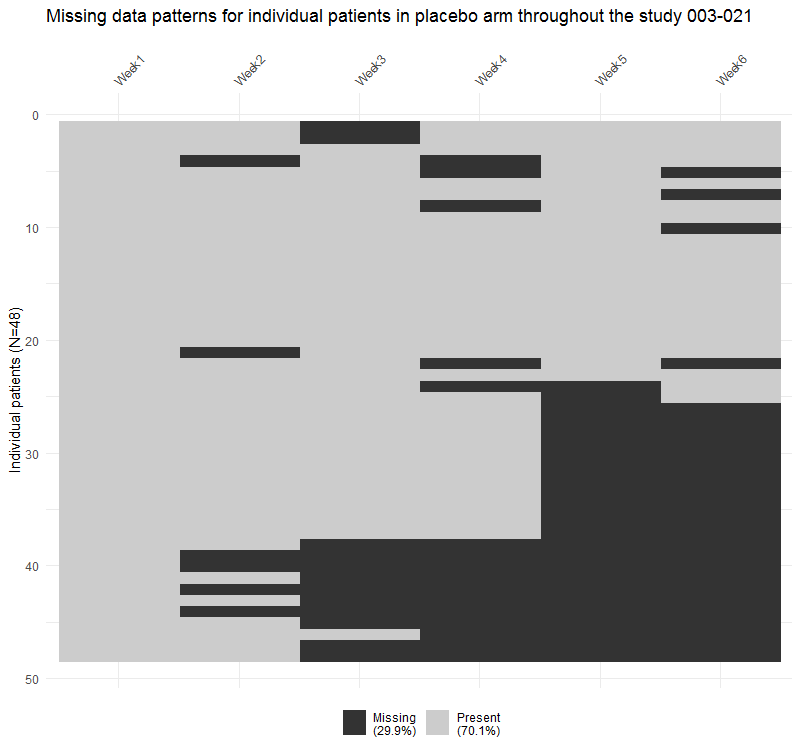

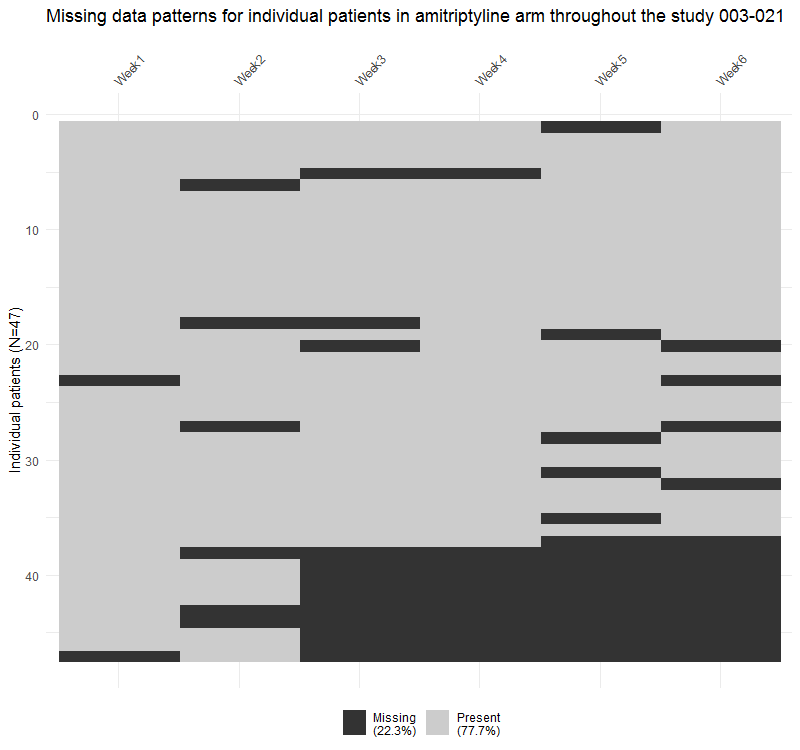


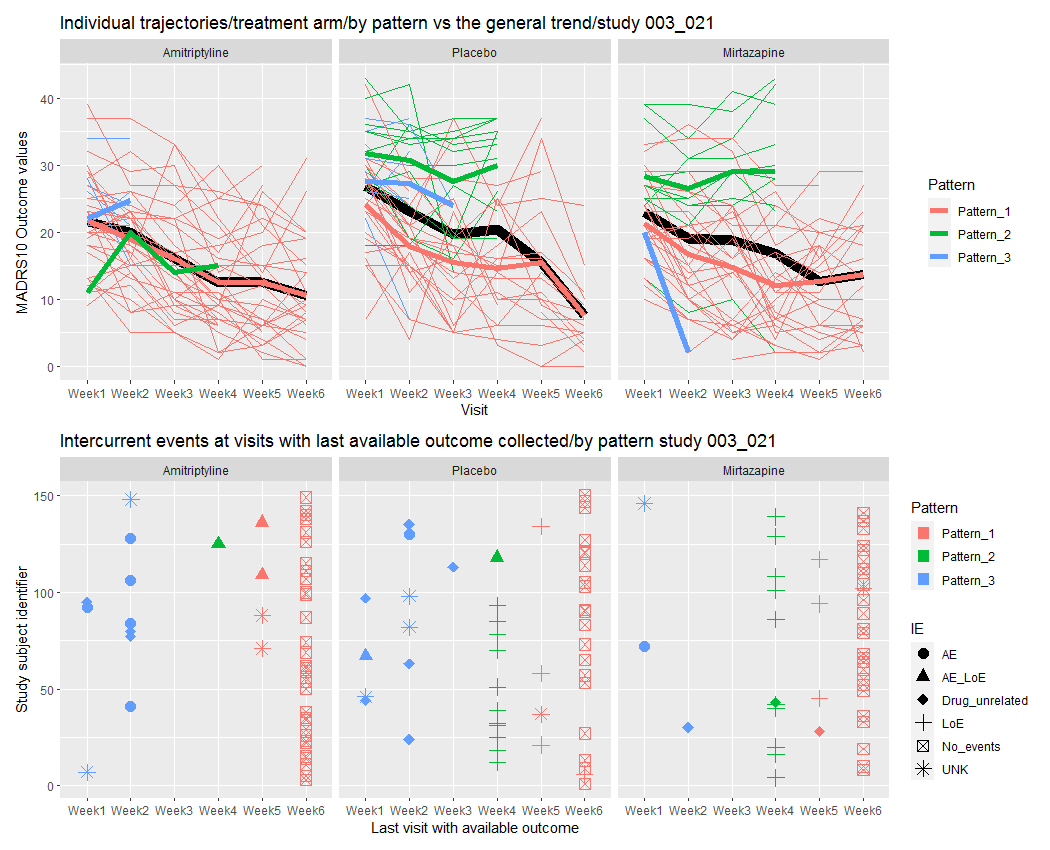


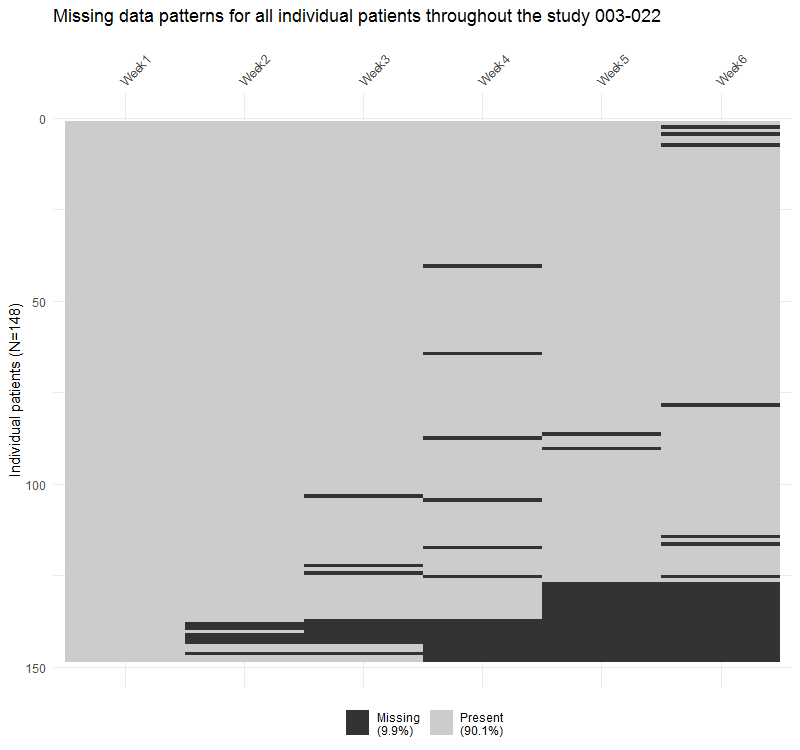


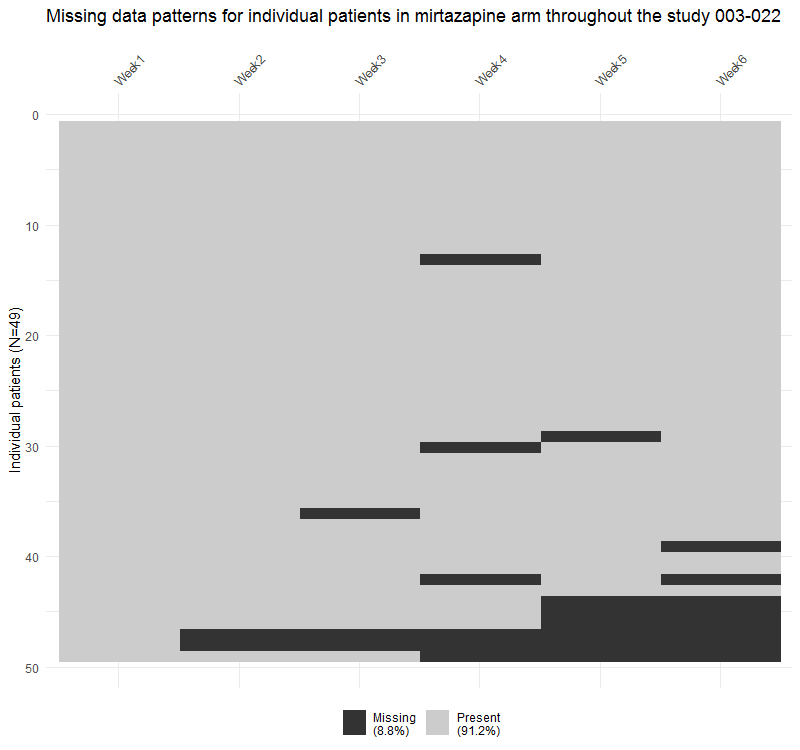

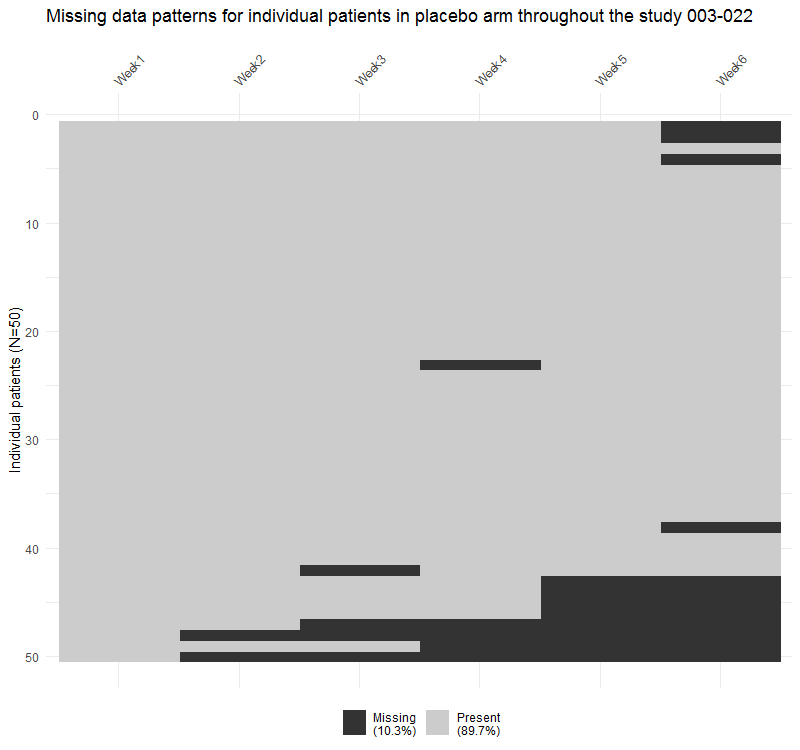

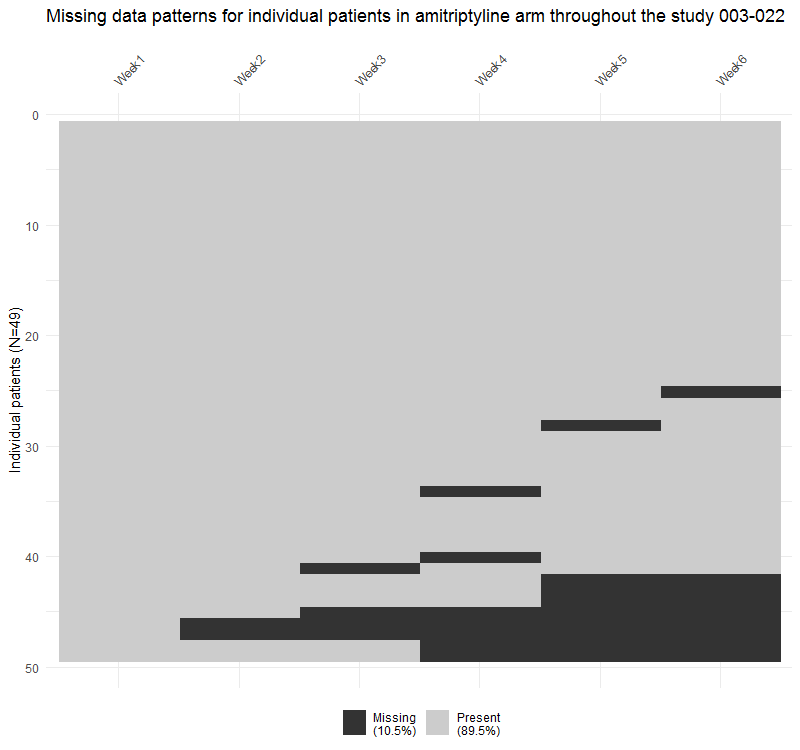


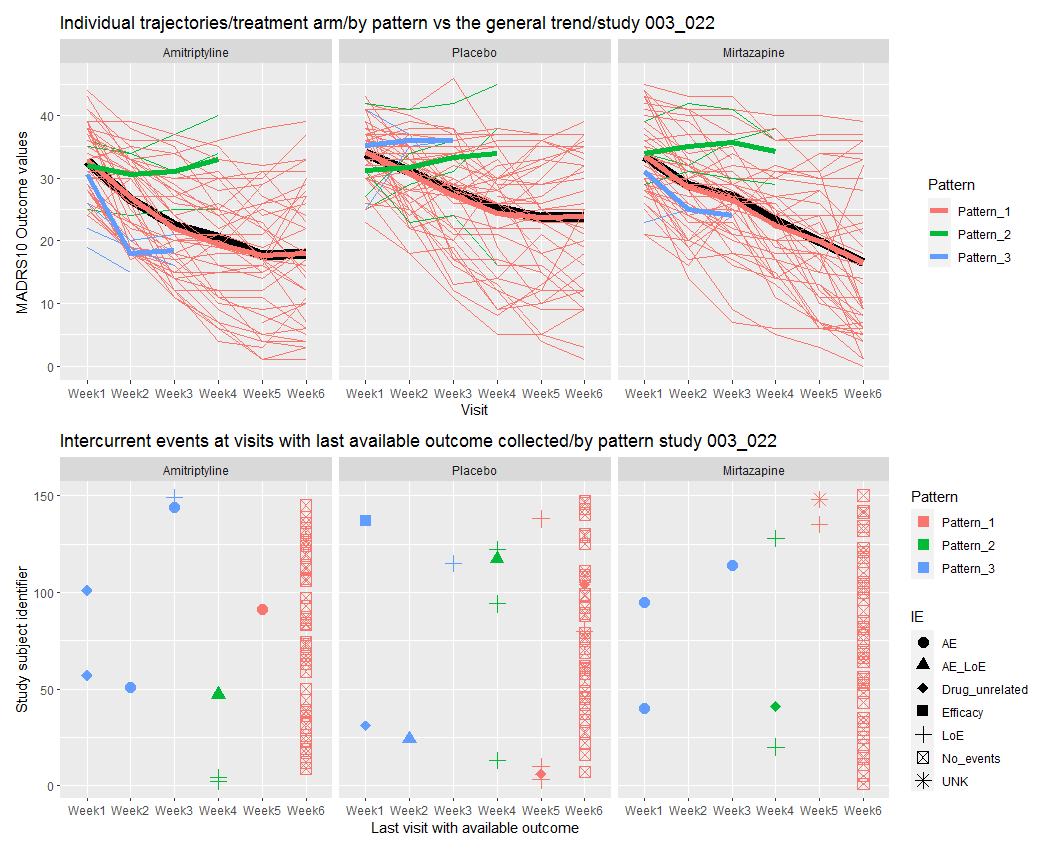

Supplement: Supplementary file 1 — Appendix S1 Supporting Information [file PST-21-1037-s001.zip › PST_2214_pst-21-0016-File009.docx]
